# Early-Life Exposure to Per- and Poly-Fluorinated Alkyl Substances and Growth, Adiposity, and Puberty in Children: A Systematic Review

**DOI:** 10.3389/fendo.2021.683297

**Published:** 2021-09-09

**Authors:** Yun Jeong Lee, Hae Woon Jung, Hwa Young Kim, Yoon-Jung Choi, Young Ah Lee

**Affiliations:** ^1^Department of Pediatrics, Seoul National University Children’s Hospital, Seoul National University College of Medicine, Seoul, South Korea; ^2^Department of Pediatrics, Kyung Hee University Medical Center, Seoul, South Korea; ^3^Department of Preventive Medicine, Seoul National University College of Medicine, Seoul, South Korea; ^4^Environmental Health Center, Seoul National University College of Medicine, Seoul, South Korea

**Keywords:** perfluorinated alkylated substances, birth weight, growth, adiposity, puberty, child, adolescent

## Abstract

Per- or polyfluoroalkyl substances (PFAS), a family of synthetic polyfluorinated compounds, are widely used in consumer products. Ubiquitous exposures to PFAS, in consideration of their persistence, bioaccumulation potential, and toxicities have led to concerns regarding possible harmful effects during critical periods of development in early-life and long-term consequences on health. The potential effects of PFAS depend on various factors including the type of PFAS and the timing and level of exposure. We performed a systematic review of the epidemiologic literature to assess the effects of early-life PFAS exposure on prenatal and postnatal growth, adiposity, and puberty in children and adolescents. For birth size, most studies indicated that prenatal PFAS exposure, in particular long-chain PFAS, may impair fetal growth, albeit some reports of null associations with maternal PFAS. For growth within 2 years of age, prenatal PFAS exposure showed no associations with height and either null or negative associations with weight. However, postnatal PFAS exposures were inversely related to height and weight at 2 years in a cross-sectional study. For postnatal adiposity, prenatal PFAS may mostly have negative associations with body mass index in the first 2 years of life, but positive relationships with adiposity in childhood and adolescence, although some studies showed null associations. For puberty, the evidence for associations between early-life PFAS exposure and pubertal development or sex hormone levels were limited and inconclusive. From experimental studies, plausible mechanisms through which PFAS may affect early-life growth and puberty include PFAS-induced activation of peroxisome proliferator-activated receptor, alterations of thyroid or steroid hormone synthesis and metabolism, and their weak estrogenic or anti-androgenic properties. Although the published literature suggests possible effects of PFAS exposures on early-life growth, adiposity, and puberty, current human evidence is limited in establishing PFAS-induced effects on early-life physical development. Further investigation is warranted to clarify PFAS-induced effects on growth and physical development in consideration of the critical time-window of exposure, concomitant exposure to chemical mixtures including various PFAS types, and possible non-monotonic dose-response relationship for growth and adiposity trajectories.

## Introduction

Per- or polyfluoroalkyl substances (PFAS) are a group of highly stable synthetic polyfluorinated compounds that exhibit unique physical and chemical characteristics, including water and oil repellency, thermal stability and surfactant properties (1). Since their first production in the 1940s and 1950s, PFAS have been incorporated into numerous products such as food packaging material, cookware, clothing, carpets, and fire extinguishers (1). By the early 2000s, PFAS became broadly distributed in the environment (2) and virtually all people living in industrialized countries were exposed to many PFAS, with blood concentrations in the ng/ml range (3).

The molecular structure of PFAS consists of a chain of carbon atoms linked to fluorine atoms. By component, PFAS are grouped into perfluoroalkyl carboxylic acids (PFCA) and perfluoroalkyl sulfonic acids (PFSA). The so called “long-chain” PFAS (PFCA with ≥ 7, and PFSA with ≥ 6 perfluorinated carbons) which includes perfluorooctanoate (PFOA), perfluorononanoic acid (PFNA), perfluorodecanoic acid (PFDA), perfluoroundecanoic acid (PFUnDA), perfluorododecanoic acid (PFDoDA), perfluorooctane sulfonic acid (PFOS), perfluoroheptanane sulfonic acid (PFHpS), and perfluorohexane sulfonic acid (PFHxS), have been reported have more bioaccumulation potential and toxicities than “short-chain” PFAS (4). Although serum levels of PFOA and PFOS, the most widely used long-chain PFAS, are declining in some countries since the early 2000s (5, 6), the use of short-chain PFAS or other novel PFAS have been increasing (7).

PFAS are resistant to environmental degradation and remain in the human body for a long time. Their half-lives are 3.8, 5.4, and 8.5 years for PFOA, PFOS, and PFHxS, respectively (8). Human exposure to PFAS may occur *via* drinking water, diet, indoor and outdoor air, house dust, and consumer products (9), and maternal PFAS can be transferred to the fetus across the placental barrier (10). Young children are likely to have higher PFAS body burdens than adults due to cumulative exposure *via* placental transfer and breastfeeding, higher water intake relative to body size, and more inhalation or ingestion of house dust due to their behavior (11).

Ubiquitous exposures to PFAS and their bioaccumulation potential have led to concerns regarding potential toxicities and health effects, particularly in growing children (12). In line with the “Developmental origins of health and diseases (DOHaD)” hypothesis (13), early-life growth and development may be more vulnerable to exposure to PFAS, with subsequent adverse health outcomes in later adulthood. We review the current epidemiologic evidence of the effects of prenatal and postnatal PFAS exposures on growth, adiposity, and puberty in children.

## Methods

This systematic review was reported according to Preferred Reporting Items for Systematic Review and meta-Analysis (PRISMA) guidelines (14).

### Definition

Exposure was defined as exposure to PFOA, PFNA, PFDA, PFUnDA, PFDoDA, perfluorotridecanoic acid (PFTrDA), perfluorotetradecanoic acid (PFTeDA), perfluorohexadecanoic acid (PFHxDA), PFOS, PFHpS, PFHxS, perfluorodecane sulfonic acid (PFDS), pentafluorobenzoic acid (PFBA), perfluoropentanoic acid (PFPeA), perfluorohexanoic acid (PFHxA), perfluoroheptanoic acid (PFHpA), perfluorobutane sulfonic acid (PFBS), perfluorooctane sulfonamide (PFOSA), N-ethyl-perfluorooctane sulfonamidoacetic acid (Et-PFOSA-AcOH), or N-methyl-perfluorooctane sulfonamidoacetic acid (Me-PFOSA-AcOH). For outcome measures, fetal growth was evaluated by measures of birth weight, birth length, ponderal index, and gestational age; postnatal growth by measures of height/length and weight; adiposity by measures of body mass index (BMI), ponderal index, waist circumference, skinfold thickness, and fat mass; pubertal outcomes by measures of pubertal stage, age at menarche, and levels of sex-hormones.

### Data Sources and Selection

We searched MEDLINE, EMBASE, Web of Science, and Scopus on May 21, 2021 with the keywords shown in **Supplementary Table 1**. The selection criteria were 1) cohort studies or case-control studies which investigated the effects of PFAS on growth, adiposity, or puberty in children and adolescents, 2) primary research articles in English and published in peer-reviewed journals. Two authors independently screened titles and abstracts using EndNote and reviewed full-text articles. Disagreements were resolved through discussion.

### Data Extraction and Risk of Bias Assessment

Two reviewers independently extracted the data including country, sample size, study design, timing of exposure, exposure measures, the level of exposure of PFAS, and outcome measures. Outcomes were expressed in terms of the odds ratio, relative risk, or β, and described in the summary of findings. We assessed the quality of the studies using the Cochrane Risk of Bias In Non-randomized Studies-of Exposure (ROBINS-E) tool (15). The preliminary template for ROBINS-E is available online (16). Disagreements in the assessment by the two authors were resolved through discussion and opinions of a third investigator were considered if needed. For risk of bias assessment, each domain of risk of bias was determined to be low, moderate, or high. Overall risk of bias was considered high if there was high risk in any of the seven domains, and low if there was low risk of bias in all seven domains.

## Results

### Study Selection

A total of 1534 articles were identified in the database. After excluding duplications (n = 993), 542 records were screened (**Supplementary Figure 1**). These articles were screened by title (376 were excluded), abstract (73 were excluded) and full text (3 were excluded). The reasons for exclusion are shown in **Supplementary Figure 1**. The 90 studies included in this review were published between November 2007 and July 2021.

### Characteristics of Included Studies

The 90 studies were comprised of 62 cohort studies, 22 cross-sectional studies, and 6 case-control studies. Sample sizes ranged from 70 to 6007. Twenty-two studies were performed in the United States, 12 in Denmark, and 8 in China. To assess for the effects of early life exposure to PFAS, 61 studies used maternal blood samples, 16 studies used cord blood samples, 11 studies used children’s blood samples, and 1 study used both maternal and childhood samples. In terms of outcomes, 49 studies evaluated for fetal growth, 5 studies for postnatal growth, 19 studies for postnatal adiposity, 6 studies for both fetal and postnatal growth or adiposity, and 11 studies for pubertal outcomes or sex-hormone levels. Among various PFAS substances, the effects of PFOA and PFOS were most frequently evaluated, followed by PFHxS and PFNA. The median exposure levels of PFOA, PFOS, PFHxS, and PFNA in maternal blood samples were ranged from 0.2-21.1 ng/mL, 0.8-33.4 ng/mL, 0.1-4.5 ng/mL, and 0.1-2.3 ng/mL, respectively. In childhood samples, the median exposure levels were 0.5-34.8 ng/mL for PFOA, 0.8-44.5 ng/mL for PFOS, 0.2-8.1 ng/mL for PFHxS, and 0.3-1.3 ng/mL for PFNA, respectively. Exposure levels for various PFAS substances of each study are described in **Supplementary Materials** (**Supplementary Tables 2–5**). The details of included articles and the results of risk of bias assessment are also shown in Supplementary materials (**Supplementary Tables 2–9**).

### Fetal Growth

The effects of prenatal PFAS exposures on fetal growth have been much studied over the past few decades. We included 52 studies for a review of birth outcomes. Prenatal exposure to PFAS was assessed during gestation from maternal blood (first to third trimester) or cord blood samples (**Table 1** and **Supplementary Table 2**). Birth outcome measures included birth weight, birth length, ponderal index, gestational age, low birth weight, small for gestational age, or preterm birth.

**Table 1:** Summary of studies assessing associations between prenatal PFAS exposure and fetal growth.

PFAS group	Timing of exposures				Outcomes			
	Early pregnancy	Mid-late pregnancy	Cord blood	Not available	Birth weight	Birth length	Ponderal index	Gestational age
PFOA					▼ (17–19); ▼ (20)^*^; ↔ (21–26)	▼ (27); ↔ (19, 22, 24–26)	↔ (27)	▲ (19); ▼ (18); ↔ (23, 24)
					▼ (17–19, 28–31); ▼ (32)^*^; ↔ (21, 22, 25, 26, 33–42); ↔ (43)^**^	▼ (28); ↔ (19, 22, 25, 26, 32, 33, 37–41); ↔ (43)^**^	▼ (37); ↔ (28, 32, 44)	▲ (19); ▼ (18, 28); ↔ (31, 32, 34, 35, 39, 41, 42)
					▼ (18, 44, 45); ↔ (21, 25, 26, 37, 46–48)	↔ (25, 26, 37, 45–48)	▼ (37, 45); ↔ (46, 47)	▼ (18); ↔ (44–46)
					↔ (49, 50)			↔ (49, 50)
PFNA					▼ (18, 23); ▼ (20)^*^; ↔ (19, 21, 22, 24, 26)	▼ (26); ↔ (19, 22, 24)		▼ (18); ▼ (23)^**^; ↔ (19, 24)
					▼ (18, 40); ▼ (38)^*^; ↔ (19, 21, 22, 26, 29, 41, 42); ↔ (43)^**^	▼ (40); ↔ (19, 22, 26, 38, 41); ↔ (43)^**^		▼ (18, 42); ↔ (19, 41)
					▼ (18); ↔ (21, 26, 44, 46, 47, 51)	↔ (26, 46, 47)	↔ (46, 47)	▼ (18); ↔ (44, 46)
PFDA					▼ (20)^*^; ↔ (18, 19, 22, 26)	▼ (26); ↔ (19, 22)		↔ (19)
					▼ (40); ▼ (38)^*^; ↔ (18, 19, 22, 26, 29, 42)	↔ (19, 22, 26, 38, 40)		↔ (19, 42)
					↔ (26, 44, 47, 51)	↔ (26, 47)	↔ (47)	↔ (44)
PFUnDA					▼ (20)^*^; ↔ (19, 26)	▼ (26); ↔ (19)		↔ (19)
					▼ (38)^*^; ↔ (19, 26, 29, 40)	↔ (19, 26, 38, 40)		↔ (19)
					↔ (26, 44, 46, 47, 51)	▼ (47)^**^; ↔ (26, 46)	↔ (46, 47)	↔ (44, 46)
PFDoDA					↔ (20, 26)	▼ (26)		
					▼ (38)^*^; ↔ (26, 29, 40)	↔ (26, 38, 40)		
					▼ (44)^*^; ↔ (26, 51)	↔ (26)		↔ (44)
PFTrDA					▼ (40)^*^	↔ (40)^*^		
PFBA					↔ (44)			↔ (44)
PFHpA					↔ (20, 26)	↔ (26)		
					↔ (26, 29)	↔ (26)		
					▼ (44)^**^; ↔ (26)	↔ (26)		↔ (44)
PFOS					▼ (18, 23); ▼ (20)^*^; ↔ (17, 19, 21, 22, 24–26)	▼ (26); ↔ (19, 22, 27, 33)	↔ (27)	▼ (18); ▼ (23)^**^; ↔ (19, 24)
					▼ (18, 31); ▼ (32, 33, 36)^*^; ▼ (43)^**^; ↔ (19, 21, 22, 26, 29, 30, 34, 35, 37, 39–42)	▼ (32)^*^; ▼ (43)^**^; ↔ (19, 26, 32, 33, 37, 39–41)	▼ (30, 37); ↔ (32)	▼ (18); ↔ (19, 31, 32, 34, 35, 39, 41, 42)
					▼ (18, 45, 46, 48); ▼ (44)^**^; ↔ (21, 26, 37, 47, 51)	▼ (48); ↔ (26, 37, 45–47)	▼ (37, 45); ↔ (46, 47)	▲ (44); ▼ (18, 46); ↔ (45)
					▼ (49, 50)			↔ (49, 50)
PFHpS					▼ (18); ↔ (19, 22)	↔ (19, 22)		▼ (18); ↔ (19)
					▼ (18); ↔ (19)	↔ (19, 22)		▼ (18); ↔ (19)
PFHxS					↔ (18–26)	↔ (19, 22, 24, 26)		↔ (18, 19, 23, 24)
					▼ (32)^*^; ↔ (18, 19, 21, 22, 26, 29, 34, 40–42); ↔ (43)^**^	▼ (32)^*^; ↔ (19, 22, 26, 40, 41); ↔ (43)^**^	↔ (32)	↔ (18, 19, 32, 34, 41, 42)
					↔ (18, 21, 26, 37, 44, 47, 51)	▲ (47)^**^	▼ (37); ↔ (47)	↔ (18, 44)
Me-PFOSA-AcOH					▼ (41); ↔ (42)	↔ (41)		↔ (41, 42)
PFBS					↔ (26)	↔ (26)		
					↔ (26)	↔ (26)		
					↔ (26)	↔ (26)		
Mixture					▼ (52)			

PFOA, perfluorooctanoic acid; PFNA, perfluorononanoic acid; PFDA, perfluorodecanoic acid; PFUnDA, perfluoroundecanoic acid; PFDoDA, perfluorododecanoic acid; PFTrDA, perfluorotridecanoic acid; PFBA, pentafluorobenzoic acid; PFHpA, perfluoroheptanoic acid; PFOS, perfluorooctane sulfonic acid; PFHpS, perfluoroheptanane sulfonic acid; PFHxS, perfluorohexane sulfonic acid; Et-PFOSA-AcOH, N-ethyl-perfluorooctane sulfonamidoacetic acid; Me-PFOSA-AcOH, N-methyl-perfluorooctane sulfonamidoacetic acid; PFBS, perfluorobutane sulfonic acid ▲, Positive associations; ▼, Inverse association; ↔, Null association; ^*^ significant only in girls; ^**^ significant only in boys.

Early and mild-late pregnancy refer to 1^st^ trimester, and 2^nd^ or 3^rd^ trimester, respectively.

Long-chain PFAS include PFOA, PFNA, PFDA, PFUnDA, and PFDoDA in perfluoroalkyl carboxylic acids (PFCA) group, and PFOS, PFHpS, PFHxS, Et-PFOSA-AcOH, and Me-PFOSA-AcOH in perfluoroalkane sulfonic acids (PFSA) group. Short-chain PFAS include PFBA and PFHpA in PFCA group, and PFBS in PFSA group. Shading boxes indicate exposure timing.

Four studies with small sample size (less than 100) (53–56), 6 studies investigating the effects of mixture of various chemicals (57–62), and 6 studies focusing on the mediating factors (63–68) are not described in the table. Further information of the included studies are described in **Supplementary Table 2**.

Studies that analyzed the relationship between PFAS exposures and birth weight have mostly shown inverse or null associations, although the results differed by sex, type of PFAS, and timing of exposure. Eleven studies reported inverse associations between PFOA exposure and birth weight (17–20, 28–32, 44, 45), while other studies showed null associations (21–26, 33–43, 46–51). Two recent meta-analyses of 16 and 24 studies (69, 70), found decreases in birth weight of 12.8 g (69) and 10.5 g (70) with 1 ng/ml increases in PFOA in maternal and cord blood, respectively. For PFOS, 14 studies showed inverse relationships with birth weight (18, 20, 23, 31–33, 36, 43–46, 48–50), while 18 showed no associations (17, 19, 21, 22, 24–26, 29, 30, 34, 35, 37, 39–42, 47, 51). A meta-analysis of nine studies showed a 0.9 g decrease in birth weight per 1 ng/ml increases in maternal PFOS exposures (69).

A few studies included analyses of long-chain PFAS other than PFOA or PFOS, such as PFNA, PFDA, PFUnDA, PFDoDA, PFTrDA, PFHpS, PFHxS, or Me-PFOSA-AcOH. Despite inconsistencies, the relationship between these long-chain PFAS and birth weight was mostly inverse (18, 20, 23, 32, 38, 40, 44), a finding which was dominant in girls (20, 32, 38, 40), while other studies showed null associations (19, 21, 22, 24–26, 29, 34, 37, 41–43, 46, 47, 51). Meanwhile, short-chain PFAS such as PFBA, PFHpA, or PFBS were only evaluated in four studies and showed no associations with birth weight (20, 26, 29, 44). Only one study assessed the effects of PFAS as mixtures using data from the Health Outcomes and Measures of Environment (HOME) study (52) and reported a small decrease in birth weight with increased exposure to a mixture of 5 PFAS (PFOA, PFNA, PFDA, PFOS, and PFHxS).

The effects of PFAS exposures on birth length, ponderal index, or gestational age were evaluated. Birth length or the ponderal index showed inverse relationships with PFOA or PFOS exposures (26–28, 30, 32, 37, 43, 45, 48) and PFNA, PFDA, PFUnDA, PFDoDA, or PFHxS exposures (26, 32, 37, 40, 47), or null associations (19, 22, 24, 38, 39, 41, 46), although one study reported a positive relationship between PFHxS exposure and birth length in boys (47). For gestational age, some studies reported increased risk for preterm birth and lower gestational age with increasing exposure to PFOA, PFNA, PFOS or PFHpS in maternal blood (18, 23, 28, 42, 46), however, no associations were demonstrated in most studies (24, 31, 32, 34, 35, 39, 41, 45, 49, 50).

Taken together, despite some inconsistency, a preponderance of studies suggest that maternal PFAS exposure, particularly to long-chain PFAS including PFOA and PFOS, negatively impact fetal growth. However, studies investigating short-chain PFAS are limited, showing no significant effects on fetal growth.

### Postnatal Growth During Infancy and Childhood

We reviewed 12 studies with outcome measures of weight, length and/or height in infancy (< 2 years) and childhood (2-12 years). In 10 studies, prenatal PFAS exposure was measured in maternal blood (during gestation or shortly after birth) or in cord blood samples taken at birth the time of birth. Two cross-sectional studies quantified exposure to PFAS in the child’s blood (71, 72). The most frequently studied PFAS was PFOA, which was quantified in all the studies that were reviewed. Many of the studies also quantified and analyzed other long-chain PFAS, such as PFOS, PFNA, PFDA, and PFHxS with high detection rates. Only a few of the studies included PFUnDA, PFDoDA, Me-PFOSA-AcOH, PFHpA, PFDS, and PFBS (**Table 2** and **Supplementary Table 3**).

**Table 2:** Summary of studies assessing associations between PFAS exposure and postnatal growth outcomes.

PFAS group		Timing of exposures					Outcomes			
							Birth to 2 years		2 to 12 years	
		Early pregnancy	Mid-late pregnancy	Maternal postpartum	Cord blood	Child	Height	Weight	Height	Weight
Prenatal exposure	PFOA						↔ (73)	↔ (73)		
							↔ (74)	▼ (74); ↔ (32, 75)	↔ (38)	↔ (38)
							↔ (76)	↔ (76)	↔ (76)	↔ (76)
							↔ (48, 77, 78)	▼ (77)^**^; ↔ (48, 78)	↔ (48)	↔ (48)
	PFNA						↔ (74)	↔ (74, 75)	↔ (38)	↔ (38)
							↔ (76)	↔ (76)	↔ (76)	↔ (76)
							↔ (77)	↔ (77)		
	PFDA							↔ (75)	▼ (38)	↔ (38)
							↔ (76)		↔ (76)	
							↔ (77)			
	PFUnDA								▼ (38)	↔ (38)
							↔ (76)	↔ (76)	↔ (76)	↔ (76)
							▲ (77)^*^	↔ (77)		
	PFDoDA							↔ (77)	▼ (38)	↔ (38)
							▲ (77)			
	PFTrDA						↔ (77)	↔ (77)		
	PFTeDA						↔ (77)	↔ (77)		
	PFHxDA						↔ (77)	↔ (77)		
	PFOS						↔ (73)	▼ (73)		
							↔ (74)	▲ (32)^*^; ▼ (74, 75)		
							↔ (76)	↔ (76)	↔ (76)	↔ (76)
							↔ (48, 77, 78)	▼ (48)^*^; ↔ (77, 78)	▲ (48)^**^	↔ (48)
	PFHxS						↔ (74)	▼ (75)^*^; ↔ (32, 74)		
							↔ (76)	↔ (76)	↔ (76)	↔ (76)
							↔ (77)	↔ (77)		
	PFDS						▼ (77)	↔ (77)		
	Me-PFOSA-AcOH							↔ (75)		
	PFBS						↔ (76)	↔ (76)		
Postnatal exposure	PFOA						▼ (71)	↔ (71)		▼ (72)
	PFNA						▼ (71)	▼ (71)		
	PFDA						▼ (71)			
	PFUnDA						↔ (71)	↔ (71)		
	PFHpA						↔ (71)	↔ (71)		
	PFOS						▼ (71)	↔ (71)	▼ (72)	
	PFHxS						▼ (71)	↔ (71)	▼ (72)	▼ (72)

PFOA, perfluorooctanoic acid; PFNA, perfluorononanoic acid; PFDA, perfluorodecanoic acid; PFUnDA, perfluoroundecanoic acid; PFDoDA, perfluorododecanoic acid; PFTrDA, perfluorotridecanoic acid; PFTeDA, perfluorotetradecanoic acid; PFHxDA, perfluorohexadecanoic acid; PFOS, perfluorooctane sulfonic acid; PFHxS, perfluorohexane sulfonic acid; PFDS, perfluorodecane sulfonic acid; Me-PFOSA-AcOH, N-methyl-perfluorooctane sulfonamidoacetic acid; PFBS, perfluorobutane sulfonic acid; PFHpA, perfluoroheptanoic acid

▲, Positive associations; ▼, Inverse association; ↔, Null association; ^*^ significant only in girls; ^**^ significant only in boys.

Early and mild-late pregnancy refer to 1^st^ trimester, and 2^nd^ or 3^rd^ trimester, respectively.

Long-chain PFAS include PFOA, PFNA, PFDA, PFUnDA, PFDoDA, PFTrDA, PFTeDA and PFHxDA in PFCA group, and PFOS, PFHxS, PFDS, and Me-PFOSA-AcOH in PFSA group. Short-chain PFAS include PFHpA in PFCA group and PFBS in PFSA group. Shading boxes indicate exposure timing.

#### Postnatal Length/Height

For early postnatal growth, there have been seven studies that analyzed for length/height outcomes by measures of postnatal length/height (in cm) or height z-scores. Six studies assessed for associations between prenatal PFAS exposures and length/height within the first 2 years of life. All of the studies that assessed for gestational PFOA and PFOS exposures by sampling maternal blood or cord blood showed null associations with measures of the child’s length after birth to 2 years (48, 73, 74, 76–78). The lack of associations to length measures was also seen in other PFAS including PFNA, PFDA, PFTrDA, PFTeDA, PFHxDA, PFHxS, PFDS, and PFBS in the same studies. The only study that reported any significant associations between prenatal exposure and postnatal length was by Cao et al., in which length at 19 months of age was positively associated with the highest tertiles of cord blood PFDoDA (in boys) and PFUnDA (in girls). However, the results were not consistent, as the same study also noted an inverse association between higher exposure to PFDS and postnatal length (77). Only one cross-sectional study, in which growth parameters was analyzed according to the children’s PFAS concentrations, showed consistent dose-response inverse associations of PFOA, PFNA, PFDA, PFOS, and PFHxS with height at 2 years of age (71).

For childhood growth past 2 years, four studies have been conducted (38, 48, 72, 76) with conflicting results. Two studies continued to assess for the effects of prenatal PFAS exposures on height measures in childhood past 2 years by including participant data up to 5 years (76) and 9 years (48) of age. While the study by Gyllenhammer et al. of the Persistent Organic Pollutants in Uppsala Primiparas (POPUP) cohort continued to show no associations between PFAS (PFOA, PFNA, PFDA, PFUnDA, PFOS, PFHxS, and PFBS) and height up to 5 years of age (76), Chen et al. noted that the height in boys of the Taiwan Birth Pnel Study (TBPS) between 24 to 60 months and between 60 and 108 months were both positively associated with cord blood PFOS (48). In contrast, Wang et al. reported inverse associations of prenatal (3^rd^ trimester) exposures to PFDA, PFUnDA and PFDoDA with height z-scores from 2 to 11 years of age in girls, without significant interactions by age (38). A cross-sectional study using data from the National Health and Nutrition Examination Survey (NHANES, 2013-2014) of the United States also reported inverse associations between PFOS and PFHxS exposures and height z-scores in children 3-11 years old (72).

Most of studies to date with regards to postnatal length/height within the first 2 years of life suggests the absence of significant associations with maternal or cord blood PFAS levels. Only one cross-sectional study demonstrated inverse associations between PFAS exposures and height at 2 years. Few studies analyzed for changes in height into later childhood and they showed conflicting associations with PFAS levels.

#### Postnatal Weight

Studies that analyzed postnatal weight have found either null or inverse associations between PFAS and measures of weight (weight-for-age, weight-for-length, and change in weight z-scores) (**Table 2** and **Supplementary Table 3**). For prenatal PFOA exposure, the majority of the studies noted no associations with weight parameters during the first 2 years of life (32, 48, 73, 75, 76, 78). However, two studies did demonstrate consistently inverse associations between PFOA and weight before 2 years of age. In a study of 334 infants with repeated anthropometric measurements from 4 weeks to 2 years of age, prenatal PFOA exposure was inversely associated with weight-for-age or weight-for-height (74). PFOA was also inversely associated with postnatal weight in boys at 19.7 ± 3.2 months of age, however the association was significant only between the second and first tertiles of PFOA (77). For PFOS, there were more studies that suggested associations with weight in the first 2 years (32, 48, 73–75) as opposed to null associations (76–78). Prenatal PFOS exposures were inversely related to weight measures in 5 month-old girls (75), in both sexes between 4 weeks and 2 years (74) and at 12 months (73). Similarly, weight z-scores in girls significantly decreased from 6 to 12 months (-0.25, 95% CI -0.47, -0.04) and 12 to 24 months (-0.24, 95% CI -0.41, -0.06) per ln-unit increase in cord blood PFOS (48). One study contrastingly showed a positive association between first trimester maternal serum PFOS and weight in girls at 20 months of age (32), however, the positive association was only significant in those born in the lower and upper birth weight stratums and not in those of the middle birthweight stratum. For all other prenatal PFAS including PFDA, PFUnDA, and PFHxS, there were no significant associations in terms of changes in weight in children before 2 years of age. However, for postnatal PFAS exposures, a cross sectional analysis of 2-year-old children found that PFOA and PFNA levels were inversely associated with weight in both sexes, although only PFNA was significant after adjustment (71). For postnatal weight in childhood past 2 years, three studies (38, 48, 76) demonstrated no associations between all prenatal PFAS exposures and childhood weight, while one cross-sectional studies showed inverse associations between PFOA and PFHxS with weight z-scores between 3-11 years of age (72).

Weight measures demonstrated more significant associations with prenatal and/or postnatal PFAS exposures, particularly in the first 2 years of life. When the results of studies that assessed for PFOA and PFOS exposures are taken together, about half of the studies show associations to weight parameters that are statistically significant. In the majority of these studies, the direction of the associations was largely negative, especially for PFOS. The evidence suggests that PFAS may have either a null effect or a negative on weight within the first two years of life, without definite associations with weight parameters after 2 years of age.

### Postnatal Adiposity

We reviewed 23 studies that examined the relationship of PFAS with measures of adiposity in infancy, childhood, adolescence and early adulthood (**Table 3** and **Supplementary Table 4**). Outcome measures varied according to age and included the ponderal index (SDS), weight gain (change in weight z-scores), body mass index (BMI, z-scores), waist circumference (z-scores, waist-to-height ratio, and/or waist-hip ratio), skinfold thickness, and fat mass as a percentage of total body mass (by air displacement plethysmography, dual-energy X-ray absorptiometry or bioelectric impedance). Prenatal exposures to PFAS were measured in maternal samples taken during gestation or shortly afterwards and in cord blood at birth. Five studies were cross-sectional, and assessed exposure and outcomes in children and adolescents between the ages of 7 and 21 years. PFOA and PFOS were the most frequently analyzed and included in all studies reviewed. More than half of the reviewed studies also analyzed for the effects of other PFAS including PFNA, PFDA, and PFHxS. PFAS that were included in some of the studies were PFDA, PFDoDA, PFHpS, PFOSA, Me-PFOSA-AcOH, PFHpA, and PFBS.

**Table 3:** Summary of studies assessing associations between PFAS exposure and adiposity outcomes.

PFAS group		Timing of exposures					Outcomes									
		Pregnancy		Maternal postpartum	Cord blood	Child	Birth to 2 years			2 to 12 years				13-20 years		
		Early	Mid-late				BMI/PI	WC	BF%	BMI	WC	SFT	BF%	BMI	WC	SFT
Prenatal exposure	PFOA						▲ (79)^*^	↔ (79)	↔ (79)	↔ (80–82)	↔ (80–82)	↔ (80)	↔ (80)			
							▼ (74, 83)		▲ (75)^**^	▲ (84); ▲ (83, 85)^†;^ ▲ (86)^*^; ▼ (87)^*^ ; ↔ (88, 89)	▲ (85, 88)^†^; ▲ (86)^*^; ▼ (87) ^*^	▲ (84)	▲ (85)^†^; ↔ (88) ; ↔ (87)^*^	▲ (90)^*^	▲ (90)^*^	
							↔ (91)			▲ (76, 91)						
							↔ (48)			↔ (48, 88, 92)	▲ (88); ↔ (92)		↔ (88, 92)			
	PFNA						▲ (79)^*^	↔ (79)	▲ (79)^*^	↔ (80, 82)	↔ (80, 82)	▲ (80)^*^	↔ (80)			
							↔ (74, 83)		▲ (75)^**^	↔ (83, 85, 88, 89); ↔ (87)^*^	↔ (85, 88); ↔ (87)^*^		↔ (85, 88); ↔ (87)^*^	↔ (90)	↔ (90)	
							↔ (91)			▲ (76); ↔ (91)						
										↔ (88, 92)	↔ (88, 92)		↔ (88, 92)			
	PFDA						▲ (79)^*^	↔ (79)	▲ (79)^*^							
									↔ (75)							
							↔ (91)			↔ (76, 91)						
										↔ (92)	↔ (92)		↔ (92)			
	PFUnDA									↔ (76)						
										↔ (92)	↔ (92)		↔ (92)			
	PFDoDA									↔ (92)	▼ (92)		▼ (92)			
	PFOS						↔ (79)	↔ (79)	↔ (79)	↔ (80–82, 86)	↔ (80–82)	↔ (80)	↔ (80)			
							▼ (74, 83)		↔ (75)	▲ (84); ▼ (83); ▼ (87)^*^ ; ↔ (85, 86, 88, 89)	▲ (86)^*^; ▼ (87)^*^ ;↔ (85, 88)	▲ (84)	↔ (85, 88); ↔ (87)^*^	↔ (90)	↔ (90)	
							▲ (91)		↔ (91)	▲ (76)						
							▼ (48)^*^			▲ (48)^*^ ;↔ (88, 92)	↔ (88, 92)		↔ (88, 92)			
	PFHxS						↔ (79)	↔ (79)	↔ (79)	↔ (80, 82)	↔ (80, 82)	▲ (80)^*^	↔ (80)			
							▼ (83) ↔ (74)		↔ (75)	▼ (83); ↔ (85, 88, 89); ↔ (87)^*^	↔ (85, 88); ↔ (87)^*^		▲ (88); ↔ (85); ↔ (87)^*^			
							↔ (91)			▲ (76); ↔ (91)						
										↔ (88, 92)	▲ (88); ↔ (92)		↔ (88, 92)			
	PFBS									↔ (92)	▲ (92)		▲ (92)			
	PFOSA													↔ (90)	↔ (90)	
	Me-PFOSA-AcOH								↔ (75)							
Postnatal exposure	PFOA									▲ (93);▼ (94)^*^; ↔ (88, 95)	▼ (94)^*^; ↔ (88, 93, 95)	↔ (95)	▼ (94)^*;^↔ (88)	↔ (96, 97); ▲ (93)	↔ (93, 96)	↔ (96)
	PFNA									↔ (88, 94)	↔ (88, 94)		↔ (88, 94)	↔ (97)		
	PFDA									▼ (94)^*^	▼ (94)^*^		↔ (94)^*^	↔ (97)		
	PFUnDA													↔ (97)		
	PFOS									▼ (94)^*^; ↔ (88, 93, 95)	▼ (94)^*^; ↔ (88, 93, 95)	↔ (95)	↔ (88) ↔ (94)^*^	▲ (96); ↔ (93, 97)	▲ (96); ↔ (93)	▲ (96)
	PFHpS													▲ (97)		
	PFHxS									↔ (88, 94)	↔ (88, 94)		↔ (88, 94)	▲ (97)		
	Me-PFOSA-AcOH									▼ (94)^*^	▼ (94)^*^		↔ (94)^*^			

BMI, body mass index; PI, ponderal index; WC, waist circumference; BF%, body fat percentage; SFT, skinfold thickness; PFOA, perfluorooctanoic acid; PFNA, perfluorononanoic acid; PFDA, perfluorodecanoic acid; PFUnDA, perfluoroundecanoic acid; PFDoDA, perfluorododecanoic acid; PFOS, perfluorooctane sulfonic acid; PFHxS, perfluorohexane sulfonic acid; PFBS, perfluorobutane sulfonic acid; PFOSA, perfluorooctane sulfonamide; Me-PFOSA-AcOH, N-methyl-perfluorooctane sulfonamidoacetic acid; PFHpS, perfluoroheptanane sulfonic acid▲, Positive associations; ▼, Inverse association; ↔, Null association; ^*^ significant only in girls; ^**^ significant only in boys; ^†^Non-monotonic dose-response relationship.

Early and mild-late pregnancy refer to 1^st^ trimester, and 2^nd^ or 3^rd^ trimester, respectively.

Long-chain PFAS include PFOA, PFNA, PFDA, and PFUnDA in PFCA group, and PFOS, PFHpS, PFHxS, PFOSA, and Me-PFOSA-AcOH in PFSA group. Shading boxes indicate exposure timing.

For infant adiposity before 2 years of age, six studies assessed various adiposity measures in infants ranging from 3 to 18 months, including the BMI (48, 74, 79, 91), ponderal index (79), trajectory of BMI changes (83), waist circumference (79), and body fat mass (75, 79, 91). For studies reporting BMI outcomes, the association to prenatal PFOA was mostly inverse (74, 83) or null (48, 91), except for one study (79). Repeated anthropometric measures between 4 weeks and 2 years showed monotonic decreases in BMI z-scores of 0.14 and 0.36 in the second and third tertiles of maternal PFOA (74) and analysis of the BMI trajectory showed that the infancy BMI zenith was lower in magnitude in the highest tertile of PFOA (83). Similarly, inverse associations between BMI and PFOS were reported in the same studies (74, 83) as well as by Chen et al., who reported decreases in BMI between 6 and 24 months in girls with increasing PFOS (48). These findings, taken together with the generally inverse associations to weight at birth and in early life, may support evidence of continued negative effects on weight by prenatal PFAS exposures. However, inconsistencies remain, as PFOS was positively associated with BMI and the risk of being overweight at 18 months of age (91), and PFOA was also positively associated with other measures of adiposity (75, 79). PFOA, along with PFNA and PFDA showed a positive association with the ponderal index in infant girls (79), and increased PFOA and PFNA were related to gains in fat mass percentage in infant boys at 5 months (75).

For BMI measures in early and mid-childhood, more than half of the studies showed positive associations with prenatal PFOA exposures. Maternal and cord blood PFOA was positively associated with higher risk of being overweight between 5 and 9 years of age (84, 86, 91), greater waist-to-height ratio (85, 86, 88), and/or BMI z-scores (76, 84) in childhood. Most of the studies that showed positive associations were those that had assessed PFOA in mid to late pregnancy, while those studies that had assessed early pregnancy PFOA showed null associations with adiposity in childhood. Only one study from the ALSPAC cohort showed inverse associations between prenatal PFOA exposures and both BMI and waist circumference in girls (87). Maternal PFOS was also modestly associated with increased BMI z-scores (76, 80, 84), waist-to-height ratio (86), skinfold thickness (84) and increased risk of being overweight/obese in childhood (84). However, other studies reported null associations with maternal PFOS (80–82, 85, 86, 88, 89, 91, 92), and one study showed inverse associations with BMI and waist circumferences in girls (87). For other prenatal PFAS exposures, PFHxS was associated with increases in the waist-to-hip ratio and the risk of overweight/obesity (88). One study from China demonstrated a positive association of PFBS, but inverse association of PFDoDA with waist circumference, waist-to-height ratio, body fat mass and body fat percentage at 5 years of age (92). For childhood exposures of PFAS and adiposity measures, one study reported consistently decreased BMI and waist-to-height ratios in 7-year old children with increased PFOA, PFDA, PFOS, and Me-PFOSA-AcOH concentrations (94), however, other studies showed null associations (88, 95).

While many studies reported positive associations of prenatal exposures PFOA and PFOS exposures with adiposity measures, non-monotonic dose-responses were noted in two studies of the HOME prospective cohort between maternal PFOA levels and adiposity measures (83, 85). Measures of BMI, waist circumference and body fat between 2 and 8 years increase up to the second tertile, followed by declines in the third tertile of PFOA (85). BMI trajectories from 4 weeks to 12 years of age showed monotonic inverse associations with PFOS and PFHxS, null associations with other prenatal PFAS, but non-monotonic dose-responses to PFOA: the timing and magnitude of the infancy BMI zenith was similar in the first and second tertiles of PFOA, followed by more rapid increases to a higher BMI between 8 and 12 years in the second tertile. The third tertile, in comparison, had an earlier and lower magnitude of the infancy BMI peak, followed by increases in absolute BMI from 8 to 12 years that were similar to the first tertile of PFOA (83).

For adiposity measures in adolescence (past 12 years of age) and young adulthood, only four studies were present including two longitudinal cohort studies that assessed exposures in maternal blood (90) or during childhood (96) and two cross-sectional study during adolescence (93, 97). Both longitudinal cohort studies showed positive associations, with Halldorsson et al., demonstrating a positive association between maternal PFOA and offspring BMI, waist circumference, and the risk of being overweight at 20 years of age (in females) (90) and Domazet et al., demonstrating that PFOS exposure at 9 years of age was positively associated with adiposity measures at 15 years (BMI, skinfold thickness and waist circumference) and at 21 years (skinfold thickness) of age (96). For cross-sectional studies, one study from NHANES showed an increased risk of overweight and/or obesity with higher PFOA exposures but not with PFOS exposures in adolescents aged 12 to 18 years (93). One other study demonstrated no definite associations between PFOA and PFOS and adolescent BMI, but significant positive relationships of PFHxS and PFHpS with the presence of overweight and obesity in adolescents aged 15 to 19 years (97).

In summary, studies of adiposity in infancy mostly support evidence for continued inverse effects of PFAS on BMI in the first 2 years of life. However, there are inconsistencies in the results, reflecting the complexities of analyzing adiposity during infancy, a time characterized by dynamic physiologic changes in weight and height (98). Studies of childhood and adolescent adiposity were split between either null or positive associations with different PFAS. For those studies that did report significant associations, the direction of the association were mostly positive for PFOA, with similar but more modest findings in regards to PFOS.

### Puberty, Menarche, and Sex Hormone Levels

Puberty is a time of transition, characterized by physiological and psychological changes, to achieve sexual maturation and fertility. The intrauterine milieu, birth size, nutrition, and endocrine disrupting chemicals (EDCs) may affect adiposity trajectory, pubertal timing and progression, with adiposity itself also contributing to changes in puberty and sex hormone levels (99–101). The relationships of PFAS exposures with pubertal onset, menarche, and sex hormone levels during childhood and adolescence have been evaluated in eleven studies (**Table 4** and **Supplementary Table 5**). The outcome measures included self-assessed pubertal stage, age at menarche, and levels of luteinizing hormone (LH), follicle stimulating hormone (FSH), testosterone, estradiol, and sex hormone-binding globulin (SHBG).

**Table 4:** Summary of studies assessing associations between PFAS exposure and puberty, menarche, and sex hormone levels.

PFAS group		Timing of exposures				Outcomes								
						Girls					Boys			
		Early pregnancy	Mild-late pregnancy	Child	Adolescent	Earlier pubertal onset	Earlier menarche	Sex hormone levels			Earlier pubertal onset	Sex hormone levels		
								Testosterone	Estradiol	Others		Testosterone	Estradiol	Others
Prenatal exposure	PFOA					↔ (102)^*^					↔ (102)			
							▼ (103); ↔ (104, 105)	▲ (106)		↔ (106)^SHBG^				
	PFNA					▲ (102)					▼ (102)^*^			
							↔ (104, 105)	↔ (106)		↔ (106)^SHBG^				
	PFDA					▲ (102)^*^					▼ (102)^*^			
							↔ (104, 105)							
	PFOS					▲ (102)^*^								
							↔ (103–105)	▲ (106)		↔ (106)^SHBG^				
	PFHpS					▲ (102)^*^					▲ (102)			
	PFHxS					▲ (102)					▲ (102)			
							↔ (104, 105)	▲ (106)		↔ (106)^SHBG^				
	PFOSA						↔ (104, 105)							
	Et-PFOSA-AcOH						↔ (104, 105)							
	Me-PFOSA-AcOH						↔ (104, 105)							
Postnatal exposure	PFOA							↔ (107)	↔ (107)			▼ (107)	↔ (107)	
							▼ (108)	↔ (107, 109–111)	↔ (107, 109, 110)	▼ (109)^SHBG^; ↔ (109)^LH,FSH^	↔ (108)	▲ (112); ↔ (109–111)	▲ (110) ↔ (109)	▲ (112)^LH^; ↔ (109)^LH,FSH,SHBG^ (112);^FSH^
	PFNA							↔ (107)	↔ (107)			↔ (107)	↔ (107)	
								↔ (109–111)	↔ (109, 110)	↔ (109)^LH,FSH,SHBG^		▼ (110) ↔ (109, 111)	↔ (109, 110)	↔ (109)^LH,FSH,SHBG^
	PFDA							↔ (110)	↔ (110)			▼ (110)	↔ (110)	
	PFUnDA							↔ (109)	↔ (109)	▼ (109)^FSH^; ↔ (109)^LH,SHBG^		↔ (109)	↔ (109)	↔ (109)^LH,FSH,SHBG^
	PFDoDA							▼ (110)	↔ (110)			↔ (110)	↔ (110)	
	PFTeDA							↔ (110)	↔ (110)			↔ (110)	↔ (110)	
	PFHxA							↔ (110)	↔ (110)			▼ (110)	↔ (110)	
	PFOS							▼ (107)	↔ (107)			▼ (107)	▼ (107)	
							▼ (108)	▼ (109),↔ (110, 111)	↔ (109, 110)	↔ (109)^LH,FSH,SHBG^	▼ (108)	▼ (110) ↔ (109, 111, 112)	↔ (109, 110)	▼ (109)^FSH^; ↔ (109)^LH,SHBG^ (112);^LH,FSH^
	PFHxS							↔ (107)	↔ (107)			↔ (107)	↔ (107)	
								↔ (110, 111)	↔ (110)			▼ (110) ↔ (111)	▲ (110)	
	PFBS							↔ (110)	↔ (110)			↔ (110)	↔ (110)	

PFOA, perfluorooctanoic acid; PFNA, perfluorononanoic acid; PFDA, perfluorodecanoic acid; PFOS, perfluorooctane sulfonic acid; PFHpS, perfluoroheptanane sulfonic acid; PFHxS, perfluorohexane sulfonic acid; PFOSA, perfluorooctane sulfonamide; Et-PFOSA-AcOH, N-ethyl-perfluorooctane sulfonamidoacetic acid; Me-PFOSA-AcOH, N-methyl-perfluorooctane sulfonamidoacetic acid; PFUnDA, perfluoroundecanoic acid; PFDoDA, perfluorododecanoic acid; PFTeDA, perfluorotetradecanoic acid; PFHxA, perfluorohexanoic acid; PFBS, perfluorobutane sulfonic acid; SHBG, sex hormone-binding globulin; LH, luteinizing hormone; FSH, follicle stimulating hormone.

▲, Positive associations; ▼, Inverse association; ↔, Null association; ^*^Non-monotonic dose-response relationship

Early and mild-late pregnancy refer to 1^st^ trimester, and 2^nd^ or 3^rd^ trimester, respectively.

Long-chain PFAS include PFOA, PFNA, PFDA, PFUnDA, PFDoDA, and PFTeDA in PFCA group, and PFOS, PFHpS, PFHxS, PFOSA, Et-PFOSA-AcOH, and Me-PFOSA-AcOH in PFSA group. Short-chain PFAS include PFHxA in PFCA group and PFBS in PFSA group. Shading boxes indicate exposure timing.

With regards to girls’ pubertal development and menarche, five studies showed inconsistent results (**Table 4**) (102–105, 108). For pubertal development, the Danish National Birth Cohort (DNBC) study recently reported substance-specific and non-monotonic associations between prenatal PFAS exposure and onset of pubertal indicators in girls (102). In detail, prenatal exposures to PFNA, PFDA, PFOS, PFHpS, and PFHxS were associated with earlier age at onset for pubertal milestones, with non-monotonic dose- responses for PFDA, PFOS, and PFHpS showing earlier pubertal indicators in the middle rather than in the highest exposure tertiles, with the lowest as a reference. For age at menarche, three prospective studies evaluated maternal exposure during pregnancy (103–105) and one cross-sectional study investigated postnatal exposure during adolescence (108). The DNBC study (103) reported later age of menarche with higher levels of prenatal PFOA exposure, while data from the Avon Longitudinal Study of Parents and Children (ALSPAC) cohort showed no associations between prenatal PFAS exposures and age at menarche (104, 105). On the contrary, the C8 Health project from United States reported a cross-sectional relationship in which higher concentrations of PFOA and PFOS were related to decreased odds of postmenarche, suggesting a later age of puberty in adolescent girls (108).

For girls’ hormone levels, five studies evaluated the relationship between PFAS exposures and testosterone, estradiol, and gonadotropin levels in girls, and the results were inconsistent (106, 107, 109–111). The ALSPAC cohort study showed positive associations between prenatal exposures to PFOA, PFOS, and PFHxS and serum testosterone levels in 15-year-old girls (106). On the contrary, three cross-sectional studies found inverse associations between postnatal PFOS (107, 109) or PFDoDA exposures (110) and serum testosterone levels in 6 to 9-year-old girls from the C8 Health project (107) or adolescent girls from Taiwan (109, 110). A cross-sectional study using data from NHANES (2011–2012) from the United States reported no relationships between PFAS exposures and testosterone levels in girls (111). Three studies showed no relationships between PFAS exposures and estradiol levels in girls during childhood and adolescence (107, 109, 110). One study reported inverse associations between PFAS exposures with FSH or SHBG levels in adolescent girls (109).

For boys’ pubertal indicators, two studies showed inconsistent results according to the timing of exposure (prenatal or postnatal) (102, 108). The aforementioned DNBC study (102) also reported substance-specific and non-monotonic associations between prenatal PFAS exposures and pubertal development in boys. In detail, prenatal PFHpS, and PFHxS exposures were associated with younger age at onset for pubertal milestones, whereas prenatal PFNA and PFDA exposures were related to later age of pubertal indicators in boys, with non-monotonous associations for PFNA and PFDA. On the contrary, the C8 Health project reported a cross-sectional relationship of reduced odds of reached puberty assessed by testosterone levels (> 50 ng/dL) with increasing PFOS levels in boys (108).

For boys’ hormone levels, five cross-sectional studies have been reported (107, 109–112). Postnatal exposure to PFOA, PFNA, PFDA, PFOS, or PFHxA showed inverse associations with serum testosterone levels (107, 110), positive associations (112), or null associations (109, 111). The cross-sectional relationships between PFAS exposures and estradiol levels were also inconsistent; positive (110), inverse (107), or null (109) according to the subjects’ age or type of PFAS. One study reported inverse associations between PFAS exposures and FSH levels in adolescent boys (109).

Overall, pediatric studies that evaluated the associations between early-life PFAS exposures and pubertal development or sex hormone levels were limited with inconsistent results according to timing, level of exposure, and type of PFAS.

## Discussions

In this review, we focused on the effects of prenatal to postnatal PFAS exposures on growth, adiposity, and puberty in children and adolescents. Contrary to relatively sufficient evidence on fetal growth and childhood adiposity, few studies have been conducted on childhood growth and puberty. Furthermore, the results were inconsistent according to sex, timing of exposure, type of PFAS, and levels of exposure. For birth size, prenatal PFAS exposure may mostly impair fetal growth. For growth within the first 2 years, prenatal PFAS exposure exhibited no association with height, but null or negative relationships with weight. However, postnatal PFAS exposure showed inverse relationship with height and weight at 2 years in a cross-sectional study. For postnatal adiposity, prenatal PFAS may mostly have negative impact on BMI in the first 2 years of life, but a positive effect on childhood and adolescent adiposity, although some studies showed no associations. For pubertal development or sex hormone levels, the evidence was limited and inconclusive.

Although mechanisms through which PFAS affect early-life growth, adiposity, and pubertal development in humans remain unclear, experimental studies have proposed potential modes of action. For prenatal and postnatal growth, one possible mechanism of PFAS toxicity is PFAS-mediated activation of the peroxisome proliferator-activated receptor α (PPARα) or PPARγ (113). Prenatal PFAS-activated PPAR signaling may alter placental and fetal metabolic tissue development, leading to impairments in fetal growth (114). In mouse models, PFOA-induced developmental toxicity and growth impairment *in utero* occurred in the presence of PPARα activity (115). Although most studies have focused on the effects of PFOA or PFOS, a recent *in-vitro* study demonstrated the human PPAR activating potential of various PFAS substances including short-chain PFAS (116). PPARs were also shown to be involved in modulating glucose and lipid metabolism or adipocyte differentiation (117). Recent epidemiologic studies showing inverse associations between maternal PFAS exposures and fatty acids during pregnancy (36) suggest a negative impact of prenatal PFAS exposures on birth weight, through reduced availability of maternal fatty acids *in utero*. Meanwhile, PFAS-induced activation of PPARγ can lead to adipogenesis and inflammation (118), contributing to increased adiposity and risk of obesity in children and adolescents.

PFAS have been identified as thyroid disrupting chemicals, potentially affecting hypothalamic-pituitary-thyroid axis, thyroid hormone synthesis and metabolism (119, 120). Since thyroid hormones play a crucial role in normal growth and development, altered thyroid function can affect early-life growth and adiposity during critical periods of development. There is a possibility that the effect of PFAS on early-life growth can be mediated by thyroid hormone disruption, although a recent study did not provide evidence of the mediating role of thyroid hormones (63). In addition, some PFAS can interfere with steroid hormone synthesis and metabolism. PFAS can inhibit 11-β hydroxysteroid dehydrogenase 2 with subsequent increases in glucocorticoid concentrations (121), leading to alterations in placental development and function, and impairment of fetal growth (122). PFOS suppresses estradiol, progesterone, and human chorionic gonadotropin secretion by placental syncytiotrophoblasts (123), which may also disrupt normal placental development and function.

For pubertal development, PFAS can affect the hypothalamic-pituitary-gonadal axis (124). PFAS can also directly affect gonads through their weak estrogenic or anti-androgenic actions (125) and perturb pubertal development. Neonatal and juvenile exposure to PFOA and PFOS in female rats altered gene expression in the kisspeptin system in the hypothalamus, leading to advanced pubertal onset (126). The effect of PFAS on childhood adiposity (127) can trigger the metabolic and peripheral signals linked to pubertal development (128). Also, PFAS can affect adrenal or gonadal steroidogenesis through altering enzyme activity (121, 129, 130).

Although experimental animal studies have suggested various plausible mechanisms, evidence in humans is still lacking. Current human evidence on the impact of PFAS exposures on childhood growth, adiposity, and puberty are limited with inconsistent data. The reasons for the discrepancies in human studies are complex, probably due to various factors, including the variety of PFAS in the environment, the developmental time-window of exposure, concomitant exposure to chemical mixtures, and the possibility of non-monotonic dose-response relationships. This complexity renders it difficult to build a reliable epidemiological model to fully reveal the mechanisms of EDC actions and to determine the real clinical impact of EDC on human health outcomes (131).

PFAS are comprised of more than 4000 individual compounds, however, previous studies mainly focused on long-chain PFAS, especially PFOA or PFOS. With the restrictions on the use of long-chain PFAS due to their persistence, bioaccumulation potential, and toxicity, they have been mainly replaced by structurally similar short-chain or other novel PFAS (132). New generation PFAS are expected to have lower toxicities due to shorter half-lives. However, the increased use of short-chain PFAS has also generated concerns regarding childhood health effects, since they have high water solubility, resistance to degradation from the environment, and modes of action similar to long-chain PFAS (7, 133). Additionally, cumulative or interactive toxicities of PFAS as mixtures remain unknown, since most studies have examined PFAS as individual compounds. A few *in vitro* studies revealed that the interactions between PFAS compounds can be complicated, as they can act additively or interact synergistically or antagonistically, depending on the species, dose ranges, duration of exposure, and mixture components (134). As humans are exposed to complex mixtures of PFAS in daily life, the combined effects or potential interactions of multiple PFAS need to be further clarified (135). Moreover, non-monotonic dose-responses, defined as a nonlinear relationship between dose and effect, are common in studies of hormones and EDCs (136). The more disruptive effect of PFAS at medium dose levels than high dose levels has been identified for childhood adiposity (83, 85) and puberty (102). Future investigation on the relationship between PFAS and health effects should include assessment of non-monotonic dose-response relationships with appropriate statistical modelling. In terms of outcomes, dynamic changes and trajectories of childhood growth, adiposity, and puberty need to be further studied with studies of prospective and longitudinal design.

## Conclusions

Early-life exposure to PFAS during critical periods of development may affect fetal and postnatal growth, adiposity, and pubertal development, potentially leading to latent health effects in adulthood. The current epidemiologic evidence has mostly suggested that prenatal PFAS exposures may impair fetal growth and increase childhood adiposity, although data on the effects of early-life PFAS exposures on childhood growth or pubertal development are still limited and inconsistent. The mechanisms through which PFAS affect early-life physical development in humans remain unclear, although experimental animal studies have suggested potential modes of action including PFAS-induced PPAR activation, altered thyroid and steroid hormone synthesis and metabolism, and weak estrogenic or anti-androgenic activity. Further research, designed to evaluate the various types of PFAS as mixtures, and in consideration of dynamic growth and adiposity trajectories, the critical time-window of exposure, and possible non-monotonic dose-response relationships, is warranted.

## Author Contributions

YL, HK, HJ, and YC performed the literature search, and wrote and revised the manuscript. YL conceptualized and coordinated the study, and critically revised the manuscript. All authors contributed to the article and approved the submitted version.

## Funding

This work was supported by the National Research Foundation of Korea (NRF) grant funded by the Korea government (MSIT) (No. 2018R1D1A1B07049806). This study was also supported by the Seoul National University Hospital Research Fund (No. 04-2018-3030).

## Conflict of Interest

The authors declare that the research was conducted in the absence of any commercial or financial relationships that could be construed as a potential conflict of interest.

## Publisher’s Note

All claims expressed in this article are solely those of the authors and do not necessarily represent those of their affiliated organizations, or those of the publisher, the editors and the reviewers. Any product that may be evaluated in this article, or claim that may be made by its manufacturer, is not guaranteed or endorsed by the publisher.
