# Supplementary material for: Early-Life Exposure to Per- and Poly-Fluorinated Alkyl Substances and Growth, Adiposity, and Puberty in Children: A Systematic Review
Source: Front Endocrinol (Lausanne). 2021 Sep 9;12:683297. doi: 10.3389/fendo.2021.683297 (PMC8458955; doi:10.3389/fendo.2021.683297)
Supplement: Supplementary file 1 [file DataSheet_1.zip › Supplementary Tables and Figure Legends.DOCX]

Supplementary Table 1. Keywords used for systematic review

| MEDLINE | ("fluorocarbons"[Title/Abstract] OR "perfluorinated"[Title/Abstract] OR "polyfluorinated"[Title/Abstract] OR "polyfluoroalkyl"[Title/Abstract] OR "perfluoroalkyl"[Title/Abstract] OR "perfluorochemicals"[Title/Abstract] OR "perfluoro compound"[Title/Abstract] OR "perfluorooctanoic acid"[Title/Abstract] OR "perfluorononanoic acid"[Title/Abstract] OR "perfluorodecanoic acid"[Title/Abstract] OR "perfluoroundecanoic acid"[Title/Abstract] OR "perfluorododecanoic acid"[Title/Abstract] OR "perfluorotridecanoic acid"[Title/Abstract] OR "perfluorotetradecanoic acid"[Title/Abstract] OR "perfluorohexadecanoic acid"[Title/Abstract] OR "perfluorooctane sulfonic acid"[Title/Abstract] OR "perfluoroheptane sulfonic acid"[Title/Abstract] OR "perfluorohexane sulfonic acid"[Title/Abstract] OR "perfluorodecane sulfonic acid"[Title/Abstract] OR "Perfluorooctane sulfonamide"[Title/Abstract] OR "N-ethyl-perfluorooctane sulfonamidoacetic acid"[Title/Abstract] OR "N-methyl-perfluorooctane sulfonamidoacetic acid"[Title/Abstract] OR "Perfluorooctane sulfonamidoacetic acid"[Title/Abstract] OR "pentafluorobenzoic acid"[Title/Abstract] OR "perfluoropentanoic acid"[Title/Abstract] OR "perfluorohexanoic acid"[Title/Abstract] OR "perfluoroheptanoic acid"[Title/Abstract] OR "perfluorobutane sulfonic acid"[Title/Abstract] OR "PFAS"[Title/Abstract] OR "PFCA"[Title/Abstract] OR "PFSA"[Title/Abstract] OR "PFOA"[Title/Abstract] OR "PFNA"[Title/Abstract] OR "PFDA"[Title/Abstract] OR "PFUnDA"[Title/Abstract] OR "PFDoDA"[Title/Abstract] OR "PFTrDA"[Title/Abstract] OR "PFTeDA"[Title/Abstract] OR "PFHxDA"[Title/Abstract] OR "PFOS"[Title/Abstract] OR "PFHpS"[Title/Abstract] OR "PFHxS"[Title/Abstract] OR "PFDS"[Title/Abstract] OR "PFOSA"[Title/Abstract] OR "PFBA"[Title/Abstract] OR "PFPeA"[Title/Abstract] OR "PFHxA"[Title/Abstract] OR "PFHpA"[Title/Abstract] OR "PFBS"[Title/Abstract]) and ("maternal exposure"[Title/Abstract] OR "prenatal exposure"[Title/Abstract] OR "fetal exposure"[Title/Abstract] OR "cord blood"[Title/Abstract] OR "fetal"[Title/Abstract] OR "infant"[Title/Abstract] OR "child"[Title/Abstract] OR "children"[Title/Abstract] OR "adolescent"[Title/Abstract]) and ("fetal development"[Title/Abstract] OR "birth weight"[Title/Abstract] OR "birth length"[Title/Abstract] OR "ponderal index"[Title/Abstract] OR "gestational age"[Title/Abstract] OR "preterm birth"[Title/Abstract] OR "small for gestational age"[Title/Abstract] OR "low birth weight"[Title/Abstract] OR "growth"[Title/Abstract] OR "body height"[Title/Abstract] OR "length"[Title/Abstract] OR "body weight"[Title/Abstract] OR "obesity"[Title/Abstract] OR "overweight"[Title/Abstract] OR "adiposity"[Title/Abstract] OR "body mass index"[Title/Abstract] OR "waist circumference"[Title/Abstract] OR "skinfold thickness"[Title/Abstract] OR "puberty"[Title/Abstract] OR "menarche"[Title/Abstract] OR "adrenarche"[Title/Abstract] OR "gonadal steroid hormones"[Title/Abstract] OR "testosterone"[Title/Abstract] OR "estradiol"[Title/Abstract]) |
| --- | --- |
| EMBASE | ('fluorocarbons':ab,ti OR 'perfluorinated':ab,ti OR 'polyfluorinated':ab,ti OR 'polyfluoroalkyl':ab,ti OR 'perfluoroalkyl':ab,ti OR 'perfluorochemicals':ab,ti OR 'perfluoro compound':ab,ti OR 'perfluorooctanoic acid':ab,ti OR 'perfluorononanoic acid':ab,ti OR 'perfluorodecanoic acid':ab,ti OR 'perfluoroundecanoic acid':ab,ti OR 'perfluorododecanoic acid':ab,ti OR 'perfluorotridecanoic acid':ab,ti OR 'perfluorotetradecanoic acid':ab,ti OR 'perfluorohexadecanoic acid':ab,ti OR 'perfluorooctane sulfonic acid':ab,ti OR 'perfluoroheptane sulfonic acid':ab,ti OR 'perfluorohexane sulfonic acid':ab,ti OR 'perfluorodecane sulfonic acid':ab,ti OR 'perfluorooctane sulfonamide':ab,ti OR 'n-ethyl-perfluorooctane sulfonamidoacetic acid':ab,ti OR 'n-methyl-perfluorooctane sulfonamidoacetic acid':ab,ti OR 'perfluorooctane sulfonamidoacetic acid':ab,ti OR 'pentafluorobenzoic acid':ab,ti OR 'perfluoropentanoic acid':ab,ti OR 'perfluorohexanoic acid':ab,ti OR 'perfluoroheptanoic acid':ab,ti OR 'perfluorobutane sulfonic acid':ab,ti OR 'pfas':ab,ti OR 'pfca':ab,ti OR 'pfsa':ab,ti OR 'pfoa':ab,ti OR 'pfna':ab,ti OR 'pfda':ab,ti OR 'pfunda':ab,ti OR 'pfdoda':ab,ti OR 'pftrda':ab,ti OR 'pfteda':ab,ti OR 'pfhxda':ab,ti OR 'pfos':ab,ti OR 'pfhps':ab,ti OR 'pfhxs':ab,ti OR 'pfds':ab,ti OR 'pfosa':ab,ti OR 'pfba':ab,ti OR 'pfpea':ab,ti OR 'pfhxa':ab,ti OR 'pfhpa':ab,ti OR 'pfbs':ab,ti) AND ('maternal exposure':ab,ti OR 'prenatal exposure':ab,ti OR 'fetal exposure':ab,ti OR 'cord blood':ab,ti OR 'fetal':ab,ti OR 'infant':ab,ti OR 'child':ab,ti OR 'children':ab,ti OR 'adolescent':ab,ti) AND ('fetal development':ab,ti OR 'birth weight':ab,ti OR 'birth length':ab,ti OR 'ponderal index':ab,ti OR 'gestational age':ab,ti OR 'preterm birth':ab,ti OR 'small for gestational age':ab,ti OR 'low birth weight':ab,ti OR 'growth':ab,ti OR 'body height':ab,ti OR 'length':ab,ti OR 'body weight':ab,ti OR 'obesity':ab,ti OR 'overweight':ab,ti OR 'adiposity':ab,ti OR 'body mass index':ab,ti OR 'waist circumference':ab,ti OR 'skinfold thickness':ab,ti OR 'puberty':ab,ti OR 'menarche':ab,ti OR 'adrenarche':ab,ti OR 'gonadal steroid hormones':ab,ti OR 'testosterone':ab,ti OR 'estradiol':ab,ti) |
| Web of Science | ("fluorocarbons" OR "perfluorinated" OR "polyfluorinated" OR "polyfluoroalkyl" OR "perfluoroalkyl" OR "perfluorochemical" OR "perfluoro compound" OR "perfluorooctanoic acid" OR "perfluorononanoic acid" OR "perfluorodecanoic acid" OR "perfluoroundecanoic acid" OR "perfluorododecanoic acid" OR "perfluorotridecanoic acid" OR "perfluorotetradecanoic acid" OR "perfluorohexadecanoic acid" OR "perfluorooctane sulfonic acid" OR "perfluoroheptane sulfonic acid" OR "perfluorohexane sulfonic acid" OR "perfluorodecane sulfonic acid" OR "Perfluorooctane sulfonamide" OR "N-ethyl-perfluorooctane sulfonamidoacetic acid" OR "N-methyl-perfluorooctane sulfonamidoacetic acid" OR "Perfluorooctane sulfonamidoacetic acid" OR "pentafluorobenzoic acid" OR "perfluoropentanoic acid" OR "perfluorohexanoic acid" OR "perfluoroheptanoic acid" OR "perfluorobutane sulfonic acid" OR "PFAS" OR "pica" OR "PFSA" OR "PFOA" OR "pcna" OR "pfoa" OR "pfundt" OR "padoda" OR "putida" OR "peteda" OR "pfhxs" OR "PFOS" OR "pfdhps" OR "pfhxs" OR "pads" OR "prosa" OR "pfoa" OR "pfmea" OR "pfhxs" OR "pfapa" OR "pcbs") and ("maternal exposure" OR "prenatal exposure" OR "fetal exposure" OR "cord blood" OR "fetal" OR "infant" OR "child" OR "children" OR "adolescent") and ("fetal development" OR "birth weight" OR "birth length" OR "ponderal index" OR "gestational age" OR "preterm birth" OR "small for gestational age" OR "low birth weight" OR "growth" OR "body height" OR "length" OR "body weight" OR "obesity" OR "overweight" OR "adiposity" OR "body mass index" OR "waist circumference" OR "skinfold thickness" OR "puberty" OR "menarche" OR "adrenarche" OR "gonadal steroid hormones" OR "testosterone" OR "estradiol") (Title) or ("fluorocarbons" OR "perfluorinated" OR "polyfluorinated" OR "polyfluoroalkyl" OR "perfluoroalkyl" OR "perfluorochemical" OR "perfluoro compound" OR "perfluorooctanoic acid" OR "perfluorononanoic acid" OR "perfluorodecanoic acid" OR "perfluoroundecanoic acid" OR "perfluorododecanoic acid" OR "perfluorotridecanoic acid" OR "perfluorotetradecanoic acid" OR "perfluorohexadecanoic acid" OR "perfluorooctane sulfonic acid" OR "perfluoroheptane sulfonic acid" OR "perfluorohexane sulfonic acid" OR "perfluorodecane sulfonic acid" OR "Perfluorooctane sulfonamide" OR "N-ethyl-perfluorooctane sulfonamidoacetic acid" OR "N-methyl-perfluorooctane sulfonamidoacetic acid" OR "Perfluorooctane sulfonamidoacetic acid" OR "pentafluorobenzoic acid" OR "perfluoropentanoic acid" OR "perfluorohexanoic acid" OR "perfluoroheptanoic acid" OR "perfluorobutane sulfonic acid" OR "PFAS" OR "pica" OR "PFSA" OR "PFOA" OR "pcna" OR "pfoa" OR "pfundt" OR "padoda" OR "putida" OR "peteda" OR "pfhxs" OR "PFOS" OR "pfdhps" OR "pfhxs" OR "pads" OR "prosa" OR "pfoa" OR "pfmea" OR "pfhxs" OR "pfapa" OR "pcbs") and ("maternal exposure" OR "prenatal exposure" OR "fetal exposure" OR "cord blood" OR "fetal" OR "infant" OR "child" OR "children" OR "adolescent") and ("fetal development" OR "birth weight" OR "birth length" OR "ponderal index" OR "gestational age" OR "preterm birth" OR "small for gestational age" OR "low birth weight" OR "growth" OR "body height" OR "length" OR "body weight" OR "obesity" OR "overweight" OR "adiposity" OR "body mass index" OR "waist circumference" OR "skinfold thickness" OR "puberty" OR "menarche" OR "adrenarche" OR "gonadal steroid hormones" OR "testosterone" OR "estradiol") (Abstract) |
| Scopus | ( TITLE ( ( "fluorocarbons" OR "perfluorinated" OR "polyfluorinated" OR "polyfluoroalkyl" OR "perfluoroalkyl" OR "perfluorochemicals" OR "perfluoro compound" OR "perfluorooctanoic acid" OR "perfluorononanoic acid" OR "perfluorodecanoic acid" OR "perfluoroundecanoic acid" OR "perfluorododecanoic acid" OR "perfluorotridecanoic acid" OR "perfluorotetradecanoic acid" OR "perfluorohexadecanoic acid" OR "perfluorooctane sulfonic acid" OR "perfluoroheptane sulfonic acid" OR "perfluorohexane sulfonic acid" OR "perfluorodecane sulfonic acid" OR "Perfluorooctane sulfonamide" OR "N-ethyl-perfluorooctane sulfonamidoacetic acid" OR "N-methyl-perfluorooctane sulfonamidoacetic acid" OR "Perfluorooctane sulfonamidoacetic acid" OR "pentafluorobenzoic acid" OR "perfluoropentanoic acid" OR "perfluorohexanoic acid" OR "perfluoroheptanoic acid" OR "perfluorobutane sulfonic acid" OR "PFAS" OR "PFCA" OR "PFSA" OR "PFOA" OR "PFNA" OR "PFDA" OR "PFUnDA" OR "PFDoDA" OR "PFTrDA" OR "PFTeDA" OR "PFHxDA" OR "PFOS" OR "PFHpS" OR "PFHxS" OR "PFDS" OR "PFOSA" OR "PFBA" OR "PFPeA" OR "PFHxA" OR "PFHpA" OR "PFBS" ) AND ( "maternal exposure" OR "prenatal exposure" OR "fetal exposure" OR "cord blood" OR "fetal" OR "infant" OR "child" OR "children" OR "adolescent" ) AND ( "fetal development" OR "birth weight" OR "birth length" OR "ponderal index" OR "gestational age" OR "preterm birth" OR "small for gestational age" OR "low birth weight" OR "growth" OR "body height" OR "length" OR "body weight" OR "obesity" OR "overweight" OR "adiposity" OR "body mass index" OR "waist circumference" OR "skinfold thickness" OR "puberty" OR "menarche" OR "adrenarche" OR "gonadal steroid hormones" OR "testosterone" OR "estradiol" ) ) OR ABS ( ( "fluorocarbons" OR "perfluorinated" OR "polyfluorinated" OR "polyfluoroalkyl" OR "perfluoroalkyl" OR "perfluorochemicals" OR "perfluoro compound" OR "perfluorooctanoic acid" OR "perfluorononanoic acid" OR "perfluorodecanoic acid" OR "perfluoroundecanoic acid" OR "perfluorododecanoic acid" OR "perfluorotridecanoic acid" OR "perfluorotetradecanoic acid" OR "perfluorohexadecanoic acid" OR "perfluorooctane sulfonic acid" OR "perfluoroheptane sulfonic acid" OR "perfluorohexane sulfonic acid" OR "perfluorodecane sulfonic acid" OR "Perfluorooctane sulfonamide" OR "N-ethyl-perfluorooctane sulfonamidoacetic acid" OR "N-methyl-perfluorooctane sulfonamidoacetic acid" OR "Perfluorooctane sulfonamidoacetic acid" OR "pentafluorobenzoic acid" OR "perfluoropentanoic acid" OR "perfluorohexanoic acid" OR "perfluoroheptanoic acid" OR "perfluorobutane sulfonic acid" OR "PFAS" OR "PFCA" OR "PFSA" OR "PFOA" OR "PFNA" OR "PFDA" OR "PFUnDA" OR "PFDoDA" OR "PFTrDA" OR "PFTeDA" OR "PFHxDA" OR "PFOS" OR "PFHpS" OR "PFHxS" OR "PFDS" OR "PFOSA" OR "PFBA" OR "PFPeA" OR "PFHxA" OR "PFHpA" OR "PFBS" ) AND ( "maternal exposure" OR "prenatal exposure" OR "fetal exposure" OR "cord blood" OR "fetal" OR "infant" OR "child" OR "children" OR "adolescent" ) AND ( "fetal development" OR "birth weight" OR "birth length" OR "ponderal index" OR "gestational age" OR "preterm birth" OR "small for gestational age" OR "low birth weight" OR "growth" OR "body height" OR "length" OR "body weight" OR "obesity" OR "overweight" OR "adiposity" OR "body mass index" OR "waist circumference" OR "skinfold thickness" OR "puberty" OR "menarche" OR "adrenarche" OR "gonadal steroid hormones" OR "testosterone" OR "estradiol" ) ) ) |

Supplementary Table 2. Studies of associations between prenatal PFAS exposure and fetal growth

| Country (year) | Sample size | Study design | Timing of exposure | Exposure matrix | Reported exposure levels (median with IQR or mean with SD) | Outcome measures | Results^*^ (β or OR with 95% CI) | References |
| --- | --- | --- | --- | --- | --- | --- | --- | --- |
| United states (2004-2005) | 293 | Cross-sectional | Cord blood | Serum | PFOA: 1.6 (1.2-2.1)  PFOS: 5 (3.5-7.9) | BW, BL, PI, GA | (i) BW (β)  PFOA: -104 (-213, 5)  PFOS: -69 (-149, 10)  (ii) PI (β)  PFOA: -0.07 (-0.138, -0.001)  PFOS: -0.074 (-0.123, -0.025) | Apelberg AJ et al. (2007) |
| Denmark (1996-2002) | 1400 | Cohort (DNBC) | Maternal (gestational week 4 and 14) | Plasma | PFOA: mean 5.6 (2.5)  PFOS: mean 35.3 (13.0) | BW, GA, SGA, LBW, preterm | (i) BW (β)  PFOA: -10.63 (-20.79, -0.47) | Fei C et al. (2007) |
| Canada (2004-2005) | 101 | Cohort (Family study) | Maternal  (gestational week 24-28, at delivery) and cord blood | Serum | PFOA: 1.8 (1.3-2.6)  PFOS: 14.5 (9.2-20.2)  PFHxS: 1.6 (1.3-2.7)  PFNA: 0.7 (0.5-0.9) | BW | No significant associations | Monroy R et al. (2008) |
| Denmark (1996-2002) | 1400 | Cohort (DNBC) | Maternal  (median gestational week 8) | Plasma | PFOA: mean 5.6 (range, <LOD-41.5)  PFOS: mean 35.3 (range, 6.4-106.7) | BL, PI | (i) BL (β)  PFOA: -0.069 (-0.113, -0.024) | Fei C et al. (2008) |
| United states (2005-2006) | 1845 (PFOA), 5262 (PFOS) | Cross-sectional (C8 Health Project) | Maternal (NA) | Serum | PFOA: 21.2 (10.3-49.8)  PFOS: 12.8 (9.0-17.7) | LBW, preterm | (i) LBW (OR)  PFOS: 1.3 (1.1, 1.6) | Stein CR et al. (2009) |
| Japan (2002-2005) | 428 | Cohort (Hokkaido Study) | Maternal (2nd-3rd trimester) | Serum | PFOA: 1.3 (0.8-1.8)  PFOS: 5.2 (3.4-7.0) | BW, BL | (i) BW (β)  PFOS (girls): -269.4 (-465.7, -73.0) | Washino N et al. (2009) |
| Canada (2005-2006) | 252 | Cohort | Maternal  (gestational week 15-16) | Serum | PFOA: 1.5 (<LOD-18)  PFOS: 7.8 (<LOD-35)  PFHxS: 0.97 (<LOD-43) | BW, BW z-score, GA, SGA, preterm | No significant associations | Hamim MP et al. (2010) |
| Norway (2003-2004) | 901 | Cohort (MoBa) | Maternal  (gestational week 17) | Plasma | PFOA: 2.2 (1.6-3.0)  PFOS: 13.0 (10.3-16.6) | BW z-score, SGA, preterm | No significant associations | Whitworth KW et al. (2011) |
| China (2007) | 167 | Cohort | Maternal (3rd trimester) | Serum | PFOA: 16.9 (14.3-21.5) | BW, BL, PI, GA, LBW, preterm | (i) BW (β)  PFOA: -267.3 (-573.3, -37.2)  (i) BL (β)  PFOA: -1.91 (-3.31, -0.52)  (i) GA (β)  PFOA: -15.99 (-27.72, -4.25) | Wu K et al. (2012) |
| Taiwan (2004) | 429 | Cross-sectional (TBPS) | Cord blood | Plasma | PFOA: GM 1.8 (2.2)  PFOS: GM 5.9 (2.0)  PFNA: GM 2.4 (4.7)  PFUnDA: GM 10.3 (3.1) | BW, BL, PI, GA, SGA, LBW | (i) BW (β)  PFOS: -110.2 (-176.0, -44.5)  (ii) BL (β)  PFNA: 0.16 (0.05, 0.27)  (iii) PI (β)  PFNA: -0.02 (-0.03, -0.004)  (iii) GA (β)  PFOS: -0.37 (-0.6, -0.13)  (iv) SGA (OR)  PFOS: 2.27 (1.25, 4.15)  (v) Preterm (OR)  PFOS: 2.45 (1.47, 4.08) | Chen M-H et al. (2012) |
| United Kingdom (1991-1992) | 447 girls | Cohort (ALSPAC) | Maternal (gestational week 15) | Serum | PFOA: 3.7 (range, 1.0-16.4)  PFOS: 19.6 (range 3.8-112.0)  PFHxS: 1.6 (range 0.2-54.8) | BW, BL, PI, GA | (i) BW (β)  PFOA:  T1: reference  T2: -56.81 (-153.05, 39.43)  T3: -133.45 (-237.37, -29.54)  PFOS:  T1: reference  T2: -111.71 (-208.24, -15.17)  T3: -140.01 (-238.14, -41.89)  PFHxS:  T1: reference  T2: -9.10 (-108.08, 89.88)  T3: -107.93 (-206.18, -9.69)  (i) BL (β)  PFOS:  T1: reference  T2: -0.72 (-1.19, -0.25)  T3: -0.63 (-1.11, -0.15)  PFHxS:  T1: reference  T2: -0.52 (-1.00, -0.04)  T3: -0.82 (-1.29, -0.34) | Maisonet M et al. (2012) |
| United states (2005-2006) | 1330 | Cross-sectional (C8 Health Project) | Maternal (NA) | Serum | PFOA: 14.3 (8.0-29.8)  PFOS: 13.9 (9.5-19.7) | BW (full-term), LBW, preterm | (i) BW (β)  PFOS (subgroup): -49 (-90, -8) | Darrow LA et al. (2013) |
| Japan (2002-2005) | 306 | Cohort (Hokkaido Study) | Maternal (2nd-3rd trimester) | Serum | PFOA: 5.6 (4.0-7.5)  PFOS: 1.4 (0.9-2.0) | BW | (i) BW (β)  PFOS (girls)  Q1: reference  Q2: -70.1 (-242.5, 102.2)  Q3: -39.1 (-216.1, 137.8)  Q4: -186.6 (-363.4, -9.8) | Kishi R et al. (2015) |
| Denmark (2008-2013) | 1507 | Cohort (Aarhus Birth cohort) | Maternal  (gestational week 9-20) | Serum | PFOA: 2.0 (1.5-2.6)  PFOS: 8.3 (6.0-10.8)  PFHxS: 0.5 (0.4-0.6)  PFNA: 0.8 (0.6-1.0)  PFDA: 0.3 (0.2-0.4)  PFHpS: 0.2 (0.1-0.2) | BW, BW z-score, BL | No significant associations | Bach CC et al. (2016) |
| South Korea (2008) | 118 | Cross-sectional | Cord blood | Serum | PFOA: 1.1 (0.8-1.3)  PFOS: 0.8 (0.6-1.0)  PFHxS: 0.6 (0.4-0.7)  PFNA: 0.4 (0.3-0.4)  PFDA: 0.1 (0.1-0.2)  PFUnDA: 0.2 (0.2-0.3)  PFDoDA: 0.1 (0.1-0.2) | BW | No significant associations | Lee E-S et al. (2016) |
| Germany (2000-2002) | 156 | Cohort (Duisburg Birth Cohort Study) | Maternal (3rd trimester) and cord blood | Plasma | PFOA: GM 2.43  PFOS: GM 9.04  PFHxS: GM 0.62 | BW, BL, PI | (i) PI (β)  PFOA: -0.41 (-0.79, -0.04)  PFOS: -0.36 (-0.70, -0.01) | Alkhalawi E et al. (2016) |
| Taiwan (2000-2001) | 223 | Cohort (Taiwan Maternal and Infant Cohort Study) | Maternal (3rd trimester) | Serum | PFOA (boys/girls)  : 2.4 (1.4-3.5) / 2.3 (1.6-3.4)  PFNA (boys/girls)  : 1.6 (0.9-2.6) / 1.6 (0.8-2.4)  PFDA (boys/girls)  : 0.5 (0.2-0.7) / 0.4 (0.2-0.6)  PFUnDA (boys/girls)  : 3.5 (1.7-9.4) / 3.3 (1.5-9.2)  PFDoDA (boys/girls)  : 0.4 (0.2-0.6) / 0.4 (0.2-0.5) | BW, BL, SGA | (i) BW (β)  PFNA (girls): -0.08 (-0.16, 0.00)  PFDA (girls): -0.14 (-0.26, -0.02)  PFUnDA (girls): -0.06 (-0.11, -0.01)  PFDoDA (girls): -0.12 (-0.21, -0.02)  (ii) SGA (OR)  PFDA (girls): 3.14 (1.07, 9.19)  PFUnDA (girls): 1.83 (1.01, 3.32) | Wang Y et al. (2016) |
| Greenland, Poland, Ukraine (2002-2004) | 1250 (Greenland 513, Poland 180, Ukraine 557) | Cohort (INUENDO) | Maternal (median gestational week 23, 25, or 33) | Serum | [Greenland] (median, 5-95th percentile)  PFOA: 1.84 (0.78-3.55)  PFOS: 20.09 (10.23-49.47)  PFNA: 0.69 (0.33-1.99)  PFDA: 0.40 (0.16-1.18)  PFUnDA: 0.70 (0.17-2.54)  PFDoDA: 0.13 (0.04-0.40)  PFHpA: 0.05 (<LOD-0.15) | BW | (i) BW (β)  PFOA: -63.8 (-122.8, -4.7 per 1.18 ng/mL) | Lenters V et al. (2016) |
| United states (1999-2002) | 1645 | Cohort (Project Viva) | Maternal (gestational week 9) | Plasma | PFOA: 5.8 (IQR, 3.8)  PFOS: 25.7 (IQR, 16.0)  PFHxS: 2.4 (IQR, 2.2)  PFNA: 0.7 (IQR, 0.4) | BW z-score, GA | (i) BW z-score (β)  PFOS: -0.04 (-0.09, 0.00)  PFNA: -0.06 (-0.11, -0.02)  (ii) GA (β)  PFOS:  (boys) -0.19 (-0.33, -0.05)  (girls) 0.01 (-0.11, 0.14)  PFNA:  (boys) -0.19 (-0.33, -0.06)  (girls) 0.03 (-0.11, 0.16)  (iii) Preterm (OR)  PFOS: -51.4 (-97.2, -5.7)  PFNA: -57.6 (-104.1, -11.2) | Sagiv SK et al. (2017) |
| Spain (2003-2008) | 1202 | Cohort (INMA) | Maternal (mean gestational week 12.3) | Plasma | PFOA: 2.35 (SD, 1.25)  PFOS: 6.05 (SD, 2.74)  PFHxS: 0.58 (SD, 0.37)  PFNA: 0.66 (SD, 0.36) | BW, BL, GA, SGA, LBW, preterm | No significant associations | Manzano-Salgado CB et al. (2017) |
| China (2013) | 321 | Cross-sectional  (Guangzhou Birth Cohort Study) | Cord blood | Serum | PFOA: 1.2 (0.9-1.8)  PFOS: 3.0 (1.7-4.6)  PFHxS: 3.9 (2.3-5.7)  PFNA: 0.2 (0.1-0.3)  PFDA: 0.1 (0.1-0.2)  PFUnDA: 0.1 (0.1-0.2)  PFDoDA: 0.1 (0.1-0.1)  PFBA: 0.1 (0.1-0.1)  PFHpA: 0.1 (0.1-0.1) | BW, GA, LBW, preterm | (i) BW (β)  PFOA: -112.7 (-171.9, -53.5)  PFOS: -95.0 (-154.0, -36.0)  PFHpA: (boys) -266.6 (-426.8, -106.3)  PFDoDA: (girls) -130.4 (-239.1, -21.7)  (i) GA (β)  PFOS: (boys) 0.29 (0.05, 0.53) | Li M et al. (2017) |
| Japan (2002-2005) | 168 | Cohort (Hokkaido Study) | Maternal (2nd-3rd trimester) | Serum | PFOA: 1.4 (0.9-2.2)  PFOS: 5.1 (3.7-6.7) | BW, PI | (i) BW (β)  PFOA: -197 (-391, -3)  (ii) PI (β)  PFOS: -2.25 (-4.01, -0.50) | Minatoya M et al. (2017) |
| China (2012) | 170 | Cross-sectional | Cord blood | Serum | PFOA: 1.1 (0.8-1.4)  PFOS: 1.0 (0.6-1.6)  PFHxS: 0.2 (0.1-0.3)  PFNA: 0.2 (0.1-0.3)  PFDA: 0.1 (0.0-0.1)  PFUnDA: 0.1 (0.0-0.1) | BW, BL, PI | (i) BL (β)  PFHxS (boys): 0.93 (0.17, 1.69)  PFUnDA (boys): -0.41 (-0.78, -0.05) | Shi Y et al. (2017) |
| Taiwan (2004-2005) | 429 | Cross-sectional (TBPS) | Cord blood | Plasma | PFOA: 2.6 (IQR, 2.4)  PFOS: 7.6 (IQR, 7.1) | BW, BL | (i) BW (β)  PFOS: -0.14 (-0.26, -0.01)  (ii) BL (β)  PFOS: -0.16 (-0.31, -0.02) | Chen M-H et al. (2017) |
| Canada (2003-2006) | 272 | Cohort (HOME study) | Maternal (gestational week 16 or 26) | Serum | PFOA: 5.4 (3.8-8.1)  PFOS: 14.4 (10-17.9)  PFHxS: 1.6 (1-2.6)  PFNA: 1.0 (0.8-1.2)  PFDA: 0.2 (0.2-0.3) | BW | No significant associations | Woods MM et al. (2017) |
| Canada (2008-2011) | 1705 | Cohort (MIREC study) | Maternal (1st trimester) | Plasma | PFOA: 1.7 (1.2-2.4)  PFOS: 4.6 (3.2-6.8)  PFHxS: 1.0 (0.7-1.6) | BW | No significant associations | Ashley-Martin et al. (2017) |
| Norway and Sweden (1986-1988) | 424 | Case-cohort | Maternal (gestational week 17-20) | Serum | Norway cohort  PFOA: 1.62 (range, 0.31-7.97)  PFOS: 9.74 (range, 0.95-59.6)  Swedish cohort  PFOA: 2.33 (range, 0.60-6.79)  PFOS: 16.4 (range, 2.28-55.2) | BW, BL, GA, SGA | No significant associations in pooled analysis | Lauritzen et al. (2017) |
| Denmark (1996-2002) | 3535 | Cohort (DNBC) | Maternal (1st and 2nd trimester) and cord blood | Plasma | PFOA: 4.6 (3.3-6.0)  PFOS: 30.1 (22.9-39.0)  PFHxS: 1.0 (0.7-1.3)  PFNA: 0.5 (0.4-0.6)  PFDA: 0.2 (0.1-0.2)  PFHpS: 0.4 (0.3-0.5) | BW, GA | (i) BW (β)  PFOA: -35.6 (-66.3, -5.0)  PFOS: -45.2 (-76.8, -13.6)  PFNA: -36.3 (-70.6, -2.0)  PFHpS: -38.9 (-72.6, -5.1)  (ii) GA (β)  PFOS: -1.1 (-1.7, -0.4)  PFNA: -1.0 (-1.7, -0.3)  PFHpS: -1.2 (-1.9, -0.5) | Meng Q et al. (2018) |
| United Kingdom (1991-1992) | 457 boys | Cohort (ALSPAC) | Maternal (median gestational week 30) | Serum | PFOA: 3.0 (2.3-3.8)  PFOS: 13.8 (11.0-17.7)  PFHxS: 1.9 (1.4-2.5)  PFNA: 0.4 (0.3-0.5) | BW, BL (crown to heal length) | (i) BW (β)  PFOS: -8.50 (-15.92, -1.07)  (ii) BL (β)  PFOS: -0.04 (-0.08, -0.01) | Marks KJ et al. (2019) |
| United States (2014-2018) | 506 | Cohort (CIOB) | Maternal (2nd trimester) | Serum | PFOA: 0.76 (0.46-1.12) PFOS: 1.93 (1.18-3.13)  PFNA: 0.30 (0.20-0.44)  Me-PFOSA-AcOH: 0.05 (0.03-0.08) | BW, BL, GA | (i) BW z-score (β)  Me-PFOSA-AcOH:  T1: reference  T2: -0.24 (-0.46, -0.02)  T3: 0.09 (-0.14, 0.32) | Eick et al. (2020) |
| Greenland (2010-2011, 2013-2016)) | 482 | Cohort (ACCEPT study) | Maternal (median gestational week 26.2 or 13) | Serum | PFOA 1.06 (range, 0.10-7.26)  PFOS 8.99 (range, 1.50-61.3)  PFHxS 0.51 (range, 0.04-4.48) PFNA 1.15 (range, 0.21-7.87)  PFDA 0.71 (range, 0.12-7.84)  PFUnDA 1.42 (range, 0.08-18.2)  PFHpS 0.16 (range, 0.06-1.44) | BW, BL, GA | (i) BW (β)  PFOA: -119 (-201, -36)  (ii) GA (β)  PFOA: 0.45 (0.17, 0.74) | Hjermitslev et al. (2020) |
| Japan (2003-2009) | 1985 | Cohort | Maternal (3rd trimester) | Serum | PFOA: 2.0 (1.3-3.3)  PFOS: 2.6 (3.4-4.7)  PFHxS: 0.3 (0.2-0.4) PFNA: 1.2 (0.9-1.6)  PFDA: (0.5 (0.4-0.7)  PFUnDA: 1.4 (1.0-1.9) PFDoDA: 0.2 (0.1, 0.2) PFTrDA: 0.3 (0.2-0.4) | BW, BL | (i) BW (β)  PFNA: -96.2 (-165.3, -27.1)  PFDA: -72.2 (-138.1, -6.3)  PFTrDA (girls) : -99.8 (-193.7, -6.0)  (ii) BL (β)  PFNA: -0.48 (-0.86, -0.11) | Kashino et al. (2020) |
| Brazil (2010-2011) | 252 (63 SGA cases and 189 controls) | Nested case-control study (BRISA) | Maternal (2nd trimester) | Whole blood | PFOA: 0.20 (0.16-0.38)  PFOS: 3.41 (2.34-5.77) | BW, preterm, SGA | (i) SGA (OR)  PFOA:  Q1: reference  Q2: 1.26 (0.54, 3.33)  Q3: 2.34 (0.94, 5.83)  Q4: 2.81 (1.12, 7.05) PFOS  Q1: reference  Q2: 0.58 (0.20, 1.67)  Q3: 0.91 (0.34, 2.43)  Q4: 3.67 (1.38, 9.74) | Souza et al. (2020) |
| Sweden (2007-2010) | 1553 | Cohort (SELMA) | Maternal (gestational week 10) | Serum | PFOA: 1.61 (1.11, 2.30)  PFOS: 5.38 (3.97, 7.60)  PFHxS: 1.23 (0.86, 1.99)  PFNA: 0.53 (0.39, 0.73)  PFDA: 0.26 (0.19, 0.34)  PFUnDA: 0.23 (0.15, 0.33)  PFHpA: 0.02 (<LOD, 0.04) | BW, BW z-score, SGA | (i) BW (β)  PFOS (girls): -85 (-145, -25)  PFOA (girls): -86 (-145, -26)  PFNA (girls): -52 (-117, -2)  PFDA (girls): -69 (-133, -6)  (ii) SGA (OR)  PFOA (girls): 1.96 (1.18, 3.28) | Wikström S et al. (2020) |
| United States (2009) | 433 | Cohort (Vanguard Pilot Study of the National Children’s Study) | Maternal (3rd trimester) | Serum | PFOA: 1.4 (0.9-2.0)  PFOS: 3.9 (2.6-5.9)  PFHxS: 0.5 (0.3-0.9)  PFNA: 0.7 (0.5-1.1)  PFDA: 0.2 (0.1-0.3)  Me-PFOSA-AcOH: ND (ND-0.2) | BW z-scores, GA, preterm birth | (i) Preterm birth (OR)  PFNA: 1.44 (1.01, 2.06) | Gardener et al. (2021) |
| China (2013-2015) | 255 | Cohort (Shanghai Birth Cohort) | Maternal  (gestational week 16, 24, 32) and cord blood | Serum | [1st trimester]  PFOA: 15.20 (11.08-20.88)  PFOS: 9.7 (6.75-15.35)  PFHxS: 0.64 (0.47-0.99)  PFNA: 2.33 (1.61-3.35)  PFDA: 1.73 (1.15-2.61)  PFUnDA: 1.43 (1.00-2.19) PFDoDA: 0.36 (0.29-0.47)  PFHpA: 0.17 (0.14-0.20)  PFBS: 0.04 (0.03-0.06) | BW, BL | (i) BL (β)  [1st trimester]  PFOS: -0.27 (-0.51, -0.02)  PFNA: -0.34 (-0.65, -0.03)  PFDA: -0.27 (-0.53, -0.01)  PFUnDA: -0.29 (-0.58, -0.01)  PFDoDA: -0.54 (-1.00, -0.08) | Chen et al. (2021) |
| Country (year) | Sample size | Study design | Timing of exposure | Exposure matrix | Exposure contrast, ng/mL (median with IQR or mean with SD) | Outcome measures | Results^*^ (β or Odds ratio with 95% CI) | References |
| Studies with sample size less than 100 | | | | | | | | |
| South Korea (2013) | 70 | Cross-sectional | Cord blood | Serum | PFOA: mean 2.09 (0.73)  PFOS: mean 3.44 (1.60)  PFHxS: mean 0.67 (0.64) | BW, BL, PI | (i) PI (OR) PFOS: 0.22 (0.05, 0.90) (ii) BW (OR) PFHxS: 0.26 (0.08, 0.85)  (iii) BL (OR) PFHxS: 0.33 (0.09, 1.17) | Lee YJ et al (2013) |
| Australia (2008~2011) | 98 | Cohort (AMETS) | Maternal (3rd trimester) | Whole blood | PFOA: 0.86 (0.21-3.1)  PFOS: 1.99 (0.45-8.1)  PFHxS: 0.33 (0.06-3.3)  PFNA: 0.30 (0.05-1.3) PFDA: 0.12 (<0.05-0.39) PFUnDA: 0.08 (<0.06-0.36) | optimal BW | (i) < 95% of optimal BW (OR)  PFHxS: 3.5 (1.1, 11.5) | Callan AC et al (2016) |
| Netherlands (2011) | 91 | Cross-sectional | Cord blood | Plasma | PFOA: 870 (200-2700) ng/L  PFOS: 1600 (570-3200) ng/L | BW | (i) BW (β) PFOS: 724.4 (193.83, 1254.97) PFOA: 168.4 (-239.18, 575.92) | de Cock M et al (2016) |
| China (2016) | 98 | Cross-sectional | Cord blood | Serum | PFOS: median 4.07  PFOA: median 1.05  6:2 CI-PFESA: median 0.73 | BW, PI, SGA | (i) BW (β) PFOS: -417.3 (742.1, -92.4) (ii) PI (β) PFOS: -0.005 (-0.008, -0.002) | Xu C et al (2019) |
| Studies regarding mixture of various chemicals | | | | | | | | |
| The Great Britain (1991~2004) | 313 | Cohort (ALSPAC) | Maternal (median gestational week 15) | Serum | [52 chemicals]  PFOA: 3.7 (2.8-4.8)  PFOS: 19.8 (15.1-24.9)  PFHxS: 1.6 (1.2-2.2)  PFNA: 0.49 (0.41-0.66)  PFDA: <LOD (<LOD-<LOD) PFOSA: 0.20 (<LOD-0.30)  Et-PFOSA-AcOH: 0.60 (0.40-0.90)  Me-PFOSA-AcOH: 0.35 (0.26-0.65) | BW | (i) BW (β) All exposure (WQS): -55g (-89, -22) | Marks KJ et al (2021) |
| Canada (2008-2011) | 1857 | Cohort (MIREC study) | Maternal (1st trimester) | Plasma | [21 chemicals]  PFOA: 1.7 (1.1-2.4)  PFOS: 4.6 (3.3-6.8)  PFHxS: 1.0 (0.7-1.6) | BW | No significant associations | Hu JMY et al (2021) |
| Belgium (2008-2009) | 248 | Cross-sectional | Cord blood | Plasma | [16 chemicals]  PFOS: GM 2.63 (95% CI, 2.45-2.83)  PFOA: GM 1.52 (95% CI, 1.44-1.61) | BW | No significant associations | Govarts E et al (2016) |
| United States (1999-2002) | 206 | Cohort (Project Viva) | Maternal (gestational week 9) | Plasma | PFOS: mean 29.1 (16.5)  PFNA: mean 0.7 (0.4) | BW | (i) BW (β) PFOS: -0.03 (-0.08, 0.02) | Rokoff LB et al (2018) |
| United States (2009-2012) | 2106 | Cohort (NICHD Fetal Growth Study) | Maternal (gestational week 10-13) | Serum | PFOA: 1.99 (1.30- 3.00)  PFOS: 5.13 (3.39- 7.98)  PFHxS: 0.71 (0.44-1.23)  PFNA: 0.76 (0.53-1.16)  PFDA: 0.25 (0.16-0.42)  PFUnDA: 0.18 (0.09-0.35)  PFDoDA: 0.03 (0.01-0.05)  PFDS: 0.01 (0.004-0.03)  PFHpA: 0.02 (0.01-0.06)  PFOSA: 0  Me-PFOSA-AcOH: 0.06 (0.03, 0.12) | BW, BL | (i) BL (β)  PFOA: -0.23 (-0.35, -0.10) | Louis et al. (2018) |
| United States (2003-2006) | 380 | Cohort (HOME study) | Maternal (gestational week 16 or 26) | Serum | [43 chemicals]  NA | BW | (i) BW (β) cluster 1: -1.2 (-1.9, -0.5)  cluster2: -0.5 (-1.1, 0.1) | Kalloo et al (2020) |
| Studies focusing on mediating factors or interaction | | | | | | | | |
| South Korea (2006-2010) | 268 | Cross-sectional (EBGRC) | Cord blood | Serum | PFOA: GM 0.8 (1.86)  PFOS: GM 0.52 (2.70)  PFHxS: GM 0.34 (1.81)  PFNA: GM 0.16 (2.14)  PFDA: GM 0.09 (1.71)  PFUnDA: GM 0.22 (1.88) PFDoDA: GM 0.07 (1.51) PFTrDA: GM 0.35 (1.88) | BW | [GSTM1 polymorphism]  (i) BW (β)  PFOA: -77.93 (-153.56, -2.30) PFOS: -49.41 (-95.57, -3.25)  PFNA: -77.02 (-135.30, -18.73)  PFDA: -101.24 (-184.80, -17.67)  PFUnDA: -83.63 (-153.94, -13.33) | Kwon EJ et al (2016) |
| Japan (2002-2005) | 177 | Cohort (Hokkaido study) | Maternal (2nd-3rd trimester) | Serum | PFOA: mean 1.6 (0.9)  PFOS: mean 5.7 (2.7) | BW, BL, PI | [DNA methylation]  (i) PI (β) PFOS: -1.07 (-1.79, -0.36) | Kobayashi S et al (2016) |
| Faroe Islands (1997-2000) | 604 | Cross-sectional | Cord blood | Serum | PFOA: 3.31 (2.54-3.99)  PFOS: 27.2 (23.1-33.1)  PFHxS: 4.54 (2.24-3.99)  PFNA: 0.59 (0.46-0.79)  PFDA: 0.28 (0.22-0.38) | BL, BW | [Gestational diabetes]  (i)BW (β)  Overall PFAS exposure  mean change per PFAS doubling: -169 (-359, 21) | Valvi D et al (2017) |
| United states (2009-2014) | 628 | Cohort (Healthy Start Study) | Maternal (median gestational week 27) | Serum | PFOA: 1.1 (0.7-1.6)  PFOS: 2.4 (1.5-3.7)  PFHxS: 0.8 (0.5-1.2)  PFNA: 0.4 (0.3-0.6)  PFDA: 0.1 (<LOD-0.2) | BW, adiposity (% fat mass) at birth | [Maternal fasting glucose]  (i) BW (β)  PFOA: -51.4 (-97.2, -5.7)  PFNA: -57.6 (-104.1, -11.2) | Starling AP et al. (2017) |
| China (2013) | 434 | Cross-sectional | Cord blood | Serum | PFOA: mean 2.64 (2.41)  PFOS: mean 1.10 (1.34) | BW, BL, PI | [Estrogen]  No significant associations | Wang H et al (2019) |
| Faroe Islands (1994-1995) | 172 | Cohort | Maternal (gestational week 34) | Serum | PFOA: GM 2.37 (GSE, 0.07)  PFOS: GM 20.86 (GSE, 0.47)  PFHxS: GM 0.55 (GSE, 0.02)  PFNA: GM 0.60 (GSE, 0.02)  PFDA: GM 0.30 (GSE, 0.01)  PFUnDA: GM 0.47 (GSE, 0.02)  PFDoDA: GM 0.03 (GSE, <0.01)  PFHpA: GM 0.03 (GSE, <0.01)  PFHpS: GM 0.35 (GSE, 0.03)  PFOSA: GM 0.04 (GSE, <0.01)  Et-PFOSA-AcOH: GM 0.65 (GSE, 0.03)  Me-PFOSA-AcOH: GM 0.18 (GSE, 0.01) | BW z-score, BL z-score | [Thyroid hormone]  (i) BW (β)  PFOS: -0.47 (-0.85, -0.04)  (ii) BL (β)  PFHxS: -0.40 (-0.72, -0.09) | Xiado C et al. (2020) |

IQR, interquartile range; SD, standard deviation; OR, odds ratio; PFOA, perfluorooctanoic acid; PFOS, perfluorooctane sulfonic acid; BW, birth weight; BL, birth length; PI, ponderal index; GA, gestational age; DNBC, Danish National Birth Cohort; SGA, small for gestational age; LBW, low birth weight; PFHxS, perfluorohexane sulfonic acid; PFNA, perfluorononanoic acid; LOD, limit of detection; NA, not available; TBPS, Taiwan Birth Panel Study; PFUnDA, perfluoroundecanoic acid; ALSPAC, Avon Longitudinal Study of Parents and Children; T1, 1st tertile; T2, 2nd tertile; T3, third tertile; Q1, 1st quartile; Q2, 2nd quartile; Q3, 3rd quartile; Q4, 4th quartile; PFDA, perfluorodecanoic acid; PFDoDA, perfluorododecanoic acid; GM, geometric mean; PFHpA, perfluoroheptanoic acid; INMA, Environment and Childhood - INfancia y Medio Ambiente; PFBA, pentafluorobenzoic acid; HOME, Health Outcomes and Measures of Environment; MIREC, Maternal-Infant Research on Environmental Chemicals; PFHpS, perfluoroheptanane sulfonic acid; CIOB, Chemicals in our Bodies; Me-PFOSA-AcOH, N-methyl-perfluorooctane sulfonamidoacetic acid; ACCEPT, Adapting to Climate Change and Environmental Pollution and Dietary Transition; PFTrDA, perfluorotridecanoic acid; BRISA, Brazilian Ribeirão Preto and São Luiz Birth Cohort Study; SELMA, Swedish Environmental Longitudinal Mother and Child, Asthma and Allergy; ND, not detected; PFBS, perfluorobutane sulfonic acid; AMETS, Australian Maternal Exposure to Toxic Substances; PFOSA, perfluorooctane sulfonamide; Et-PFOSA-AcOH, N-ethyl-perfluorooctane sulfonamidoacetic acid; WQS, weighted quantile Sum; NICHD, National Institute of Child Health and Human Development; EBGRC, Ewha Birth & Growth Retrospective Cohort; GSE, geometric standard error

^*^ Statistically significant findings are described.

Supplementary Table 3. Studies assessing associations between PFAS exposure and postnatal growth outcomes

| Country (year) | Sample size | Study design | Timing of exposure | Exposure matrix | Reported exposure levels (median with IQR or mean with SD) | Outcome measures | Results^*^ (β or OR with 95% CI) | References |
| --- | --- | --- | --- | --- | --- | --- | --- | --- |
| Denmark (1996-2002) | 1010 | Cohort (DNBC) | Maternal (1st trimester) | Plasma | PFOA: 5.21 (IQR, 3.06)  PFOS: 33.4 (IQR, 17.2) | Weight, length, and BMI at 5 and 12 months of age | (i) Weight at 12mo  PFOS -5.8 (-10.4, -1.2) | Andersen et al. (2010) |
| United Kingdom (1991-1992) | 447 girls | Cohort (ALSPAC) | Maternal (median gestational week 15) | Serum | PFOA: 3.7 (range, 1.0-16.4)  PFOS: 19.6 (range 3.8-112.0)  PFHxS: 1.6 (range 0.2-54.8) | Weight at 20 months | (i) Weight (β)  PFOS:  T1: reference  T2: 310.64 (27.19, 594.08)  T3: 579.82 (301.40, 858.25) | Maisonet et al. (2012) |
| Netherland (2011-2013) | 89 | Cohort | Cord blood | Plasma PFOA, PFOS | PFOA 0.87 (range, 0.3-2.7)  PFOS 1.61 (range, 0.57-3.20) | Weight, length, and BMI (1, 2, 4, 6, 9, 11 months) | No significant associations | Cock et al. (2014) |
| Taiwan (2000-2001) | 223 | Cohort (Taiwan Maternal and Infant Cohort Study) | Maternal (3rd trimester) | Serum | PFOA (boys/girls)  : 2.4 (1.4-3.5) / 2.3 (1.6-3.4)  PFNA (boys/girls)  : 1.6 (0.9-2.6) / 1.6 (0.8-2.4)  PFDA (boys/girls)  : 0.5 (0.2-0.7) / 0.4 (0.2-0.6)  PFUnDA (boys/girls)  : 3.5 (1.7-9.4) / 3.3 (1.5-9.2)  PFDoDA (boys/girls)  : 0.4 (0.2-0.6) / 0.4 (0.2-0.5) | Weight and height at ages 2, 5, 8, and 11. | (i) Height z-score (β)  PFNA (boys): -0.15 (-0.37, 0.08) (significant only at ages 8 and 11)  PFDA (girls): -0.52 (-0.80, -0.24)  PFUnDA (girls): -0.14 (-0.27, -0.01)  PFDoDA (boys): -0.17 (-0.40, 0.06) (significant only at ages 8 and 11)  (girls): -0.25 (-0.49, 0.00) | Wang et al. (2016) |
| Taiwan (2004-2005) | 429 | Cohort (TBPS) | Cord blood | Plasma | PFOA: 2.6 (IQR, 2.4)  PFOS: 7.6 (IQR, 7.1) | Weight, length/height, and BMI at 4, 6, 12, 24, 60, 84, and 108 months. | (i) Weight z-score (β)  PFOS (girls): -0.25 (-0.47, -0.04) at 6-12 months; -0.24 (-0.41, -0.06) at 12-24 months  (ii) Height z-score (β)  PFOS (boys): 0.18 (0.03, 0.33) at 24-60 months; 0.19 (0.01, 0.38) at 60-108 months  (iii) BMI z-score (β)  PFOS (girls): -0.33 (-0.59, -0.08) at 6-12 months; -0.25 (-0.45, -0.05) at 12-24 months; 0.34 (0.007, 0.68) at 60-108 months | Chen et al. (2017) |
| Sweden (1996-2011) | 200 | Cohort (POPUP cohort) | Maternal (3 weeks after delivery) | Serum | PFOA: mean 2.4 (SE, 0.06) ng/g  PFOS: mean 14 (SE, 0.44) ng/g  PFHxS: mean 3.6 (SE, 0.20) ng/g  PFNA: mean 0.46 (SE, 0.01) ng/g  PFDA: mean 0.23 (SE, 0.007) ng/g  PFUnDA: mean 0.19 (SE, 0.006) ng/g  PFBS: mean 0.03 (SE, 0.003) ng/g | Weight and length at 3, 6, 12, and 18 months and weight, height, and BMI at ages 3, 4, and 5 years. | NA (Exact data points not specified. Results are shown as figures) | Gyllenhammer et al. (2018) |
| United States (2003-2006) | 229 | Cohort (HOME study) | Maternal (gestational week 16 or 26, or at birth) | Serum | PFOA: 5.5 (3.8-7.7) PFOS: 14 (9.6-18)  PFHxS: 1.5 (NA)  PFNA: 0.9 (NA) | Repeated weight, length, weight-for-length, BMI between 4 weeks and 2 years of age. | (i) Weight-for-age z-score (β)  PFOA:  T1: reference  T2: -0.02 (-0.34, 0.29)  T3: -0.43 (-0.78, -0.14)  PFOS:  T1: reference  T2: 0.01 (-0.31, 0.32)  T3: -0.33 (-0.65, -0.01)  (ii) Weight for length z-score (β)  PFOA:  T1: reference  T2: -0.31 (-0.53, -0.06)  T3: -0.34 (-0.59, -0.08)  PFOS:  T1: reference  T2: -0.16 (-0.41, 0.09)  T3: -0.31 (-0.56, -0.05)  (iii) BMI z-score (β)  PFOA:  T1: reference  T2: -0.14 (-0.38, 0.10)  T3: -0.36 (-0.60, -0.12)  PFOS:  T1: reference  T2: -0.03 (-0.27, 0.21)  T3: -0.32 (-0.56, -0.07) | Shoaff et al. (2018) |
| China (2013-2015) | 282 | Cohort (Zhoukou city birth cohort) | Cord blood | Serum | PFOA: 1.25 (0.87- 1.82)  PFOS: 1.01 (0.60-1.76)  PFHxS: 0.09 (0.05-0.15)  PFNA: 0.11 (0.07-0.16)  PFDA: 0.10 (0.06-0.15)  PFUnDA: 0.08 (006-0.12)  PFDoDA: 0.02 (0.01-0.04)  PFTrDA: <LOD  PFTeDA: <LOD  PFHxDA: <LOD  PFDS: <LOD | Weight, length and ponderal index (at mean 19.7±3.2 months) | (i) Weight in grams (β)  PFOA (boys)  T1: reference  T2: -661 (-1194, -128)  T3: -285 (-831, 262)  (ii) Length in cm (β)  PFDoDA (boys)  T1: reference  T2: 0.37 (-2.02, 2.75)  T3: 2.44 (0.15, 4.73)  PFUnDA (girls)  T1: reference  T2: 3.01 (0.13, 5.89)  T3: 3.37 (0.25, 6.49)  PFDS (boys and girls)  Below LOD: reference  Above LOD: -2.87 (-5.31, -0.44) | Cao et al. 2018 |
| South Korea (2012-2013) | 361 | Cross-sectional (EDC cohort) | Childhood (2 years) | Serum | PFOA: mean 5.02 (3.04)  PFOS: mean 5.27 (3.38)  PFHxS: mean 1.40 (0.89)  PFNA: mean 2.03 (2.48)  PFDA: mean 0.40 (0.24)  PFUnDA: mean 0.47 (0.32)  PFHpA: mean 0.45 (0.48) | Weight, height, BMI  Changes in weight and height (from birth to age 2) | (i) Weight in kg (β)  PFNA: -0.32 (-0.48, -0.15)  (ii) Weight change from birth in kg (β)  PFNA: -0.26 (-0.44, -0.10)  (iii) Height in cm (β)  PFOA: -0.91 (-1.36, -0.47)  PFOS: -0.77 (-1.27, -0.15)  PFHxS: -0.84 (-1.26, -0.42)  PFNA: -0.48 (-1.40, -0.51)  PFDA: -0.44 (-0.77, -0.10)  (iv) Height gain from birth in cm (  β)  PFOA: -0.86 (-1.52, -0.20)  PFOS: -0.71 (-1.27, -0.15)  PFHxS: -0.89 (-1.45, -0.33) | Lee et al. (2018) |
| United States (2009-2014) | 402 | Cohort (Healthy Start Study) | Maternal (median gestational week 27) | Serum | PFOA: 1.0 (0.7- 1.6)  PFOS: 2.2 (1.4-3.4)  PFNA: 0.4 (0.2-0.6)  PFDA: 0.1 (0.1-0.2)  PFHxS: 0.7 (0.5-1.2)  Me-PFOSA-AcOH: 0.1 (<LOD-0.1) | Infant weight, length and adiposity (fat mass and fat free mass by air displacement plethysmography) at 5 months. Rapid weight gain defined as weight z-score gain >0.67SD. | (i) Weight for age (z-score) (β)  PFOS (girls): -0.26 (-0.43, -0.10)  PFHxS (girls): -0.17 (-0.33, -0.01)  (ii) Weight for length (z-score) (β)  PFOS (girls): -0.24 (-0.43, -0.05)  (iii) Infant adiposity (β)  PFOA (boys): 1.53 (0.35, 2.71)  PFNA (boys): 1.67 (0.56, 2.78)  (iv) Rapid growth in weight for age (z-score) (OR)  Me-PFOSA-AcOH  <LOD: reference  >LOD: 2.17 (1.09, 4.13)  (v) Rapid growth in weight for length (z-score)  PFHxS (β): 1.49 (1.02, 2.18)  Me-PFOSA-AcOH (OR):  <LOD: reference  >LOD: 3.31 (1.75, 6.25) | Starling et al. (2019) |
| Sweden (2007-2010) | 1334 | Cohort (SELMA) | Maternal (mean gestational week 10) | Serum | PFOA: mean 1.6 (1.8) | Trajectory of weight from birth to age 5.5 years (birth; week 2; months 2, 3, 4, 5, 6, 8, 10, and 12; years 1.5, 2.5, 3, 4, 5.5) characterized by: infant slope (kg/month), infant peak growth velocity (kg/month), age at infant peak growth velocity (months), infant weight plateau (kg), infant growth spurt duration (months) | (i) Age of infant peak growth velocity (months) (β)  PFOA (girls): 1.01 (0.38, 1.65)  (ii) Infant weight plateau (kg) (β)  PFOA (girls): 1.37 (0.47, 2.27) | Tanner et al. (2020) |
| United States (2013-2014) | 600 | Cross-sectional (NHANES) | Childhood (3-11 years) | Serum | PFOA GM 1.92 (SE, 0.08)  PFOS GM 3.90 (SE, 0.17)  PFNA GM 0.80 (SE, 0.06)  PFHxS GM 0.85 (SE, 0.04) | Height, weight, and BMI z-scores (3-11 years) | (i) Height z-score  PFOS  T1 reference  T2 -0.32 (-0.60, -0.04)  T3 -0.39 (-0.72, -0.06)  PFHxS  T1 reference  T2 -0.32 (-0.68, 0.04)  T3 -0.41 (-0.63, -0.19)  Mixture  T1 reference  T2 -0.27 (-0.55, 0.01)  T3 -0.42 (-0.69, -0.154)  (ii) Weight z-score  PFOA  T1 reference  T2 -0.33 (-0.63, -0.04)  T3 -0.28 (-0.65, 0.08)  PFHxS  T1 reference  T2 -0.30 (-0.67, 0.07)  T3 -0.42 (-0.76, -0.08)  PFOS  T1 reference  T2 -0.32 (-0.60, -0.04)  T3 -0.40 (-0.76, -0.04)  Mixture  T1 reference  T2 -0.32 (-0.63, -0.00)  T3 -0.37 (-0.66, -0.08) | Scinicariello et al. (2020) |

IQR, interquartile range; SD, standard deviation; OR, odds ratio; DNBC, Danish National Birth Cohort; PFOA, perfluorooctanoic acid; PFOS, perfluorooctane sulfonic acid; BMI, body mass index; ALSPAC, Avon Longitudinal Study of Parents and Children; PFHxS, perfluorohexane sulfonic acid; T1, 1st tertile; T2, 2nd tertile; T3, third tertile; PFNA, perfluorononanoic acid; PFDA, perfluorodecanoic acid; PFUnDA, perfluoroundecanoic acid; PFDoDA, perfluorododecanoic acid; TBPS, Taiwan Birth Panel Study;

POPUP, Persistent Organic Pollutants in Uppsala Primiparas; SE, standard error; PFBS, perfluorobutane sulfonic acid; NA, not available; HOME, Health Outcomes and Measures of Environment; PFTrDA, perfluorotridecanoic acid; LOD, limit of detection; PFTeDA, perfluorotetradecanoic acid; PFHxDA, perfluorohexadecanoic acid; perfluorodecane sulfonic acid; EDC, Environment and Development of Children; PFHpA, perfluoroheptanoic acid; Me-PFOSA-AcOH, N-methyl-perfluorooctane sulfonamidoacetic acid; SELMA, Swedish Environmental Longitudinal Mother and Child, Asthma and Allergy; NHANES, National Health and Nutrition Examination Survey; GM, geometric mean

^*^ Statistically significant findings are described.

Supplementary Table 4. Studies assessing associations between PFAS exposure and adiposity outcomes

| Country (year) | Sample size | Study design | Timing of exposure | Exposure matrix | Reported exposure levels (median with IQR or mean with SD) | Outcome measures | Results^*^ (β or OR with 95% CI) | References |
| --- | --- | --- | --- | --- | --- | --- | --- | --- |
| Denmark (1988-1989) | 665 | Cohort (Aarhus Birth Cohort) | Maternal (gestational week 30) | Serum | PFOA: 3.7 (IQR, 2.0)  PFOS: 21.5 (IQR, 9.1)  PFNA: 0.3 (IQR, 0.2)  PFOSA: 1.1 (IQR, 1.0) | BMI and waist circumference measures (423 from clinical examination; 242 self-reported from web-based questionnaire) at 20 years of age | (i) BMI (β)  PFOA  Q1: reference  Q4: 1.6 (0.6, 2.6)  (ii) Waist circumference (β)  PFOA  Q1: reference  Q4: 4.3 (1.4, 7.3) | Halldorsson et al. (2012) |
| Denmark (1996-2002) | 811 | Cohort (DNBC) | Maternal (median gestational week 8) | Plasma | PFOA: 5.25 (IQR, 2.99)  PFOS: 33.8 (IQR, 17.6) | BMI, waist circumference, risk of being overweight at 7 years of age | No significant associations | Andersen et al. (2013) |
| Denmark (1997) | 499 | Cross-sectional (EYHS) | Childhood (8-10 years) | Plasma | PFOA: 9.3 (range, 0.8-35.2)  PFOS: 41.5 (range, 6.2-132.5) | BMI, waist circumference, and skinfold thickness | No significant associations | Amalie G et al. (2014) |
| Greenland and Ukraine (2002-2004) | 1022 (Greenland 531, Ukraine 491) | Cohort (Birth cohorts of Greenland and Kharkiv, Ukraine) | Maternal (mean gestational week 24 | Plasma | [Greenland]  PFOA: 1.8 (range, 0.5-5.1)  PFOS: 20.2 (range, 4.1-87.3)  [Ukraine]  PFOA: 1.0 (range, 0.2-9.8)  PFOS: 5.0 (range 0.7-18.1) | BMI, waist circumference (waist-to-height ratio > 0.5), risk of being overweight between 5 to 9 years of age (median 8.3 years in Greenland and 7.0 years in Ukraine) | (i) Overweight (RR)  PFOA:  T1 reference  T3 1.81 (1.04,3.17) in Greenlandic girls  (ii) Waist-to-height ratio>0.5 (RR)  PFOA:  T1 reference  T2 1.93 (1.15, 3.24) in Greenlandic girls  PFOS (continuous):  1.38 (1.05, 1.82) in girls from both countries | Høyer et al. (2015) |
| United States (2003-2006) | 204 | Cohort (HOME study) | Maternal (gestational week 16, or 26, or at birth) | Serum | PFOA: 5.3 (3.7-7.7)  PFOS: 13.0 (9.1-18.0)  PFNA 0.9 (0.7-1.2)  PFHxS 1.4 (0.8-2.3) | BMI, waist circumference, body fat percent (bioelectric impedance) at ages 2,3,4,5 and 8 years. | (i) BMI z-score at 8 years (β)  PFOA  T1 reference  T2 0.44 (0.13, 0.74)  T3 -0.01 (-0.33, 0.30)  (ii) waist circumference at 8 years (β)  PFOA  T1 reference  T2 4.3 (1.7, 6.9)  T3 2.2 (-0.5, 4.9)  (iii) Body fat percent difference (β)  T1 reference  T2 3.6 (1.8, 5.5)  T3 1.5 (-0.4, 3.4)  (iv) Overweight/obesity (RR)  T1 reference  T2 1.84 (0.97, 3.50)  T3 1.54 (0.77, 3.07) | Braun et al. (2016) |
| Denmark (1997) | 501 (9 years), 444 (15 years), 369 (21 years) | Cohort (EYHS study) | Childhood (9, 15, and 21 years) | Plasma | [At 9 years]  PFOA: (boys/girls) 9.7 (7.7-12.1) / 9.0 (7.4-11.2)  PFOS: (boys/girls) 44.5 (35.4-55.7) / 39.9 (34.3-49.3)  [At 15 years]  PFOA: (boys/girls) 3.7 (2.7-4.4) / 3.4 (2.8-4.5)  PFOS: (boys/girls) 22.3 (16.5-25.1) / 20.8 (15.9-24.7)  [At 21 years]  PFOA: (boys/girls) 3.1 (2.5-3.9) / 2.7 (2.1-3.4)  PFOS: (boys/girls) 11.9 (9.2-15.2) / 9.1 (7.0-10.8) | BMI, waist circumference, skinfold thickness at ages 9, 15, and 21 | NA (Exact data points not specified. Results are shown as figures) | Domazet et al. (2016) |
| United States (1999-2002) | 1006 (in early childhood), 876 (in mid childhood) | Cohort (Project Viva) | Maternal (median gestational week 9) | Plasma | [Children with early childhood data]  PFOA: 5.6 (4.1-7.7)  PFOS: 24.8 (18.4-34.1)  PFHxS: 2.4 (1.6-3.8)  PFNA: 0.6 (0.5-0.9)  [Children with mid childhood data]  PFOA: 5.6 (3.9-7.6)  PFOS: 24.7 (18.2-33.6)  PFHxS: 2.3 (1.6-3.7)  PFNA: 0.6 (0.5-0.9) | BMI, waist circumference (waist-to-hip ratio), skinfold thickness in early (age 3.2) and mid (age 7.7) childhood. DXA total fat mass and fat free mass indexes and DXA trunk fat mass index in mid childhood. | (i) BMI (β)  PFOS: 0.28 (0.02,0.55) in girls  PFNA 0.30 (0.06, 0.55) in girls  (ii) Sum of subscapular and triceps skinfold thickness (β)  PFHxS: 0.57 (0.03,1.10) in girls  PFNA: 1.01 (0.16, 1.86) in girls  (iii) Subscapular to triceps skinfold thickness ratio (β)  PFHxS: 1.61 (0.58, 2.65) in girls  PFNA: 2.17 (0.52, 3.83) in girls | Mora et al. (2016) |
| Faroe Islands (2007-2009) | 444 (at 18 months), 349 (at age 5) | Cohort (National Hospital of the Faroe Islands birth cohort) | Maternal (2 weeks after delivery) | Serum | PFOA: GM 1.37 (IQR, 0.95-1.95)  PFOS: GM 8.04 (IQR, 6.23-10.6)  PFHxS: GM 0.19 (IQR 0.13-0.31)  PFNA: GM 0.67 (0.52-0.86)  PFDA: GM 0.26 (0.19-0.35) | BMI at 18 months and age 5 | (i) BMI-z score at 18 months (β)  PFOS: 0.23 (0.04, 0.42)  (ii) Overweight at age 5 (RR)  PFOA: 1.50 (1.01,2.24) | Karlsen et al. (2017) |
| Spain (2003-2008) | 1154 (at 6 months), 1230 (at age 4), 1086 (at age 7) | Cohort (INMA) | Maternal (median gestational week 12.7) | Plasma | PFOA: GM 2.32  PFOS: GM 5.80  PFHxS: GM 0.61  PFNA: GM 0.66 | Gain in weight z-score from birth to 6 months   BMI and waist circumference (z-scores, waist-to-height ratio) at ages 4 and 7. | (i) Weight gain z-score (birth to 6 months) (β)  PFOA 0.13 (0.01, 0.26) in boys | Manzano-Salgado et al. (2017) |
| United Kingdom (1991-1992) | 359 girls | Cohort (ALSPAC) | Maternal (gestational week 16) | Serum | PFOA: 3.7 (2.9-4.8)  PFOS: 19.7 (15.0-25.3)  PFHxS: 1.7 (1.3-2.2)  PFNA: 0.5 (0.4-0.7) | BMI, waist circumference, and body fat percent (DXA) at 9 years of age | (i) BMI (β)  PFOA: -0.16 (-0.32, 0.00)  PFOS: -0.04 (-0.07, 0.00)  (ii) Waist circumference (β)  PFOA: -0.54 (-0.97, -0.11)  PFOS: -0.12 (-0.20, -0.04) | Hartman et al. (2017) |
| Norway and Sweden (1986-1988) | 412 (137 SGA births and 275 non-SGA births) | Cohort (The SGA Study) | Maternal (gestational week 17-20) | Serum | [Norway]  PFOA 1.64 (0.82-3.54)  PFOS: 9.62 (3.78-24.6)  [Sweden]  PFOA: 2.33 (0.95-4.28)  PFOS: 16.3 (7.17-30.5) | BMI, skinfold thickness and risk of overweight/obesity at age 5 | (i) BMI for age z-scores (β)  PFOA: 0.32 (0.05, 0.60) for Norway only  PFOS: 0.18 (0.01, 0.35) for all  (ii) Triceps skinfold z-score (β)  PFOA: 0.24 (0.05, 0.42) for Norway only  PFOS: 0.15 (0.02, 0.27) for all  (iii) Overweight (OR)  PFOA: 2.90 (1.10,7.63) for Norway only  PFOS: 2.04 (1.11, 3.74) for all | Lauritzen et al. (2018) |
| China (2012-2017) | 404 | Cohort (Shanghai Prenatal Cohort) | Cord blood | Plasma | PFOA: 6.74 (range, 0.7-29.97)  PFOS: 2.44 (range, 0.39-65.61)  PFHxS: 0.16 (range, 0.07-0.85)  PFNA: 0.64 (range, 0.18-3.29) PFDA: 0.36 (range, <LOD-2.51) PFUnDA: 0.40 (range, <LOD-5.73)  PFDoDA: 0.09 (range, <LOD-0.62)  PFBS: 0.05 (range, <LOD-0.39) | BMI, waist circumference, waist-to-height ratio, fat mass, and body fat percent, at 5 years of age | PFBS  (i) Waist circumference (β)  PFDoDA  T1: reference  T2: -1.95 (-3.61, -0.3)  PFBS  T1: reference  T3: 2.06 (0.43, 3.68)  (ii) waist-to-height ratio  PFBS (continuous): 0.01 (0.0008, 0.03)  (iii) Fat mass (kg) (β)  PFDoDA  T1: reference  T2: -0.93 (-1.65, -0.2)  PFBS  T1: reference  T3: 0.79 (0.08, 1.51) (iv) Body fat % (β)  PFDoDA  T1: reference  T2: -3.02 (-5.61, -0.43)  PFBS (continuous) 2.84 (0.29, 5.39) | Chen et al. (2019) |
| United states (2004-2006) | 353 | Cross-sectional (Cincinnati cohort of the BCERP) | Childhood (mean age 7.1; range 6-8.8) | Serum | PFOA: mean 9.49  PFOS: mean 15.87  PFHxS: mean 9.10  PFNA: mean 1.53  PFDA: mean 0.30  Me-PFOSA-AcOH: mean 1.23 | BMI, waist circumference (waist-to-height ratio, waist-hip ratio) total fat mass and fat mass percent (Tanita fat mass), and bioelectrial resistance | (i) BMI z-score (β)  PFOA: -0.33  PFOS: -0.31  PFDA: -0.36  Me-PFOSA-AcOH: -0.18  (ii) Waist-to-height ratio (β)  PFOA: -0.0079  PFOS: -0.0101  PFDA: -0.0131  Me-PFOSA-AcOH: -0.0060  (iii) Fat mass (β)  PFOA: -0.23  (iv) Fat mass (%) (β)  PFOA: -2.64 | Fassler et al. (2019) |
| Sweden (2003-2008) | 1048  (354 overweight cases vs. 64 controls) | Case-control  (Southern Sweden Maternity Cohort) | Maternal (gestational week 14) | Serum | PFOA: 3.1 (2.4-4.2)  PFOS: 16.6 (12.6-22.0)  PFHxS: 0.7 (0.5-1.0)  PFNA: 0.4 (0.3-0.5) | Cases defined by age and sex adjusted BMI ≥18 kg/m^2^ (corresponding to a BMI of 25 kg/m2 for adults). | No significant associations | Martinsson et al (2020) |
| Denmark (2010-2012) | 612 (at birth), 602 (at 3.2 months) and 530 (at 19.2 months) | Cohort (Odense Child Cohort) | Maternal (gestational week <16) | Serum | (median, 5-95th percentile)  PFOA: 1.62 (0.67-4.03)  PFOS: 8.04 (3.82-15.46)  PFHxS: 0.30 (0.08-0.66)  PFNA: 0.66 (0.33-1.52)  PFDA: 0.23 (0.15-0.53) | BMI, ponderal index, waist circumference, and skinfold thickness (to calculate body fat percentage) at 3 and 18 months. | (i) Ponderal index (β)  PFOA: 0.07 (0.01, 0.13)  PFNA: 0.24 (0.08, 0.41)  PFDA: 0.60 (0.18,1.02)  (ii) BMI SDS (β)  PFNA: 0.18 (0.02, 0.34)  PFDA: 0.42 (0.01, 0.84) | Jensen et al (2020) |
| United States (2003-2006) | 212 | Cohort (HOME study) | Maternal (gestational week 16 or 26), cord blood,  Childhood 3, 8, 12 years | Serum | (i) PFOA  Maternal: 5.3 (3.7-7.2)  Cord blood: 3.2 (2.4-4.7) 3yr: 5.4 (3.7-7.5) 9yr: 2.5 (1.8-3.2) 12yr: 1.3 (1.0-1.7)  (ii) PFOS  Maternal: 13.3 (9.0-18.0) Cord blood: 4.2 (3.2-6.4) 3yr: 6.2 (4.5-9.7)  8yr: 3.6 (2.7-4.7)  12yr: 2.4 (1.8-3.2)  (iii) PFNA  Maternal: 0.9 (0.7-1.2)  Cord blood: 0.4 (0.3-0.6) 3yr: 1.3 (1.0-1.8) 8yr: 0.7 (0.5-1.1) 12yr: 0.3 (0.3-0.5)  (iv) PFHxS  Maternal: 1.3 (0.8-2.3)  Cord blood: 0.6 (0.4-1.1)  3yr: 1.9 (0.9-3.3)  8yr: 1.2 (0.9-1.8)  12yr: 0.7 (0.5-1.0) | BMI, waist circumference, waist-to-hip ratio, body fat percent, fat mass index (DXA) at 12 years of age | (i) Overweight/obesity (by fat mass index) (OR)  Maternal PFHxS: 1.71 (1.08, 2.73)  (ii) Waist-to-hip ratio (β)  Maternal PFOA: 0.02 (0.00, 0.03)  Cord blood PFOA: 0.02 (0.00, 0.03) Cord blood PFHxS: 0.02 (0.00, 0.03) | Liu et al. (2020) |
| United States (2003-2006) | 345 | Cohort (HOME study) | Maternal (gestational week 16, or 26, or at birth) | Serum | PFOA: 5.5 (3.8-7.7)  PFOS: 13.8 (9.6-18.1)  PFNA: 0.9 (0.7-1.0)  PFHxS: 1.5 (0.9-2.3) | Longitudinal BMI measurements at 4 weeks, ages 1-5, 8, and 12. BMI trajectories characterized by infancy BMI zenith (magnitude and age), BMI nadir (magnitude and age), velocity of BMI increase from age 8-12, and absolute BMI at 12. | (I) BMI from 4 weeks to 12 years of age  (i) Magnitude of infancy BMI zenith (β)  PFOS: -0.4 (-0.75, -0.06)  PFHxS: -0.42 (-0.8, -0.06)  (ii) Magnitude of BMI nadir (β)  PFOS: -0.67 (-1.02, -0.19)  PFHxS: -0.60 (-1.09, -0.09)  (iii) BMI at 12 years (β)  PFOS: -1.41 (-2.65, -0.14)  PFHxS: -0.50 (-1.78, 0.76) | Braun et al (2021) |
| Norway (2010-2011) | 940 | Case-control | Adolescence (mean age 16.5, range 15-19) | Serum | PFOA: GM 2.14 (IQR, 1.26)  PFOS: GM 5.71 (IQR, 2.64)  PFHxS: GM 0.80 (IQR, 0.53)  PFNA: GM 0.61 (IQR, 0.40)  PFDA: GM 0.27 (IQR, 0.20)  PFUnDA: GM 0.17 (IQR, 0.13)  PHFpS: GM 0.14 (IQR, 0.06) | Cases defined as overweight (BMI >1SD) and/or obese (BMI >2SD) | (i) Obesity (OR)  PFHxS  Q1: reference  Q3: 2.71 (1.17,6.25)  PFHpS  Q1: reference  Q2: 2.73 (1.05,7.05)  Q3: 2.84 (1.10, 7.37) | Averina et al (2021) |
| United States (1999-2000, 2003-2012) | 2473 | Cross-sectional (NHANES) | Adolescence (12-18 years) | Serum | PFOA: mean 3.79 (SE, 0.14)  PFOS: mean 15.66 (SE, 0.65) | BMI (overweight and/or obesity), waist circumference (abdominal obesity) | Overweight/obesity (OR)  PFOA  Q1: refernce  Q2: 1.42 (0.85, 2.38) Q3: 2.22 (1.20, 4.13)  Q4: 2.73 (1.10, 6.74) | Geiger et al. (2021) |

IQR, interquartile range; SD, standard deviation; OR, odds ratio; PFOA, perfluorooctanoic acid; PFOS, perfluorooctane sulfonic acid; PFNA, perfluorononanoic acid; PFOSA, perfluorooctane sulfonamide; BMI, body mass index; DNBC, Danish National Birth Cohort; EYHS, European Youth Heart Study; RR, relative risk; HOME, Health Outcomes and Measures of Environment; PFHxS, perfluorohexane sulfonic acid; T1, 1st tertile; T2, 2nd tertile; T3, third tertile; NA, not available; DXA, dual-energy X-ray absorptiometry; GM, geometric mean; PFDA, perfluorodecanoic acid; INMA, Environment and Childhood - INfancia y Medio Ambiente; ALSPAC, Avon Longitudinal Study of Parents and Children; SGA, Scandinavian Successive Small-for-Gestational Age births; LOD, limit of detection; PFUnDA, perfluoroundecanoic acid; PFDoDA, perfluorododecanoic acid; PFBS, perfluorobutane sulfonic acid; BCERP, Cincinnati cohort of the Breast Cancer and Environment Research Program; Me-PFOSA-AcOH, N-methyl-perfluorooctane sulfonamidoacetic acid; SDS, standard deviation score; PFHpS, perfluoroheptanane sulfonic acid; Q1, 1st quartile; Q2, 2nd quartile; Q3, 3rd quartile; Q4, 4th quartile; NHANES, National Health and Nutrition Examination Survey

^*^ Statistically significant findings are described.Supplementary Table 5. Studies assessing associations between PFAS exposure and puberty, menarche, and sex hormone levels

| Country (year) | Sample size | Study design | Timing of exposure | Exposure matrix | Reported exposure levels (median with IQR or mean with SD) | Outcome measures | Results^*^ (β or OR with 95% CI) | References |
| --- | --- | --- | --- | --- | --- | --- | --- | --- |
| Puberty, menarche | | | | | | | | |
| United Kingdom (1991-1992) | 448 girls (218 cases with menarche < 11.5 years of age vs. 230 control) | Nested Case-control study (ALSPAC) | Maternal (gestational week 16) | Serum | PFOA: 3.7 (2.8-4.8)  PFOS: 19.8 (15.1-24.9)  PFHxS: 1.6 (1.2-2.2)  PFNA: 0.6 (0.5-0.8)  Et-PFOSA-AcOH: 0.6 (0.4-0.9)  Me-PFOSA-AcOH: 0.4 (0.3-0.8) | Earlier age of menarche (< 11.5 years) by questionnaire at 13 years of age | No significant associations | Christensen  et al. (2011) |
| United States (2005-2006) | 6007 (3076 boys, 2931 girls) | Cross-sectional  (C8 Health Project) | Childhood (8-18 years) | Serum | PFOA: (boys/girls) median 26/20  PFOS: (boys/girls) median 20/18 | Indicators of sexual maturation: self-reported menarche status or sex steroid hormone measurements (boys, total T > 50 ng/dL or free T > 5 pg/mL; girls, E2 > 20 pg/mL) | (I) Boys (OR)  (i) PFOS  Q1: reference  Q3: 0.58 (0.37, 0.91)  Q4: 0.46 (0.29, 0.72)  (II) Girls (OR)  (i) PFOA  Q1: reference  Q2: 0.58 (0.37, 0.90)  Q3: 0.55 (0.35, 0.86)  (ii) PFOS  Q1: reference  Q3: 0.61 (0.38, 0.96) | Lopez-Espinosa et al. (2011) |
| Denmark (1988-1989) | 343 girls | Cohort (Aarhus Birth Cohort) | Maternal (gestational week 30) | Serum | PFOA: 3.6 (2.8-4.8)  PFOS: 21.1 (16.7-25.5) | Age of menarche by questionnaire | (i) PFOA (β)  T1: reference  T2: 0.9 (-3.0, 4.8)  T3: 5.3 (1.3, 9.3) | Kristensen et al. (2013) |
| Denmark (1999-2003) | 1167 (591 boys, 576 girls) | Cohort (Puberty Cohort nested within DNBC) | Maternal (gestational week 9) | Plasma | (sample 1/sample 2)  PFOA: 4.8 (2.7-8.2) / 4.1 (2.3-6.4)  PFOS: 32.3 (19.3-50.8) / 27.9 (16.5-42.2)  PFHxS: 1.0 (0.6-.6)  PFNA: 0.5 (0.3-0.8)  PFDA: 0.2 (0.1-0.3)  PFHpS: 0.4 (0.2-0.6) | Pubertal status (including Tanner stage) by questionnaires (every six months, from 11.5 years of age until Tanner V or 18 years of age) | Average differences in the age of puberty  (i) Boys  PFHxS  T1: reference  T3: -6.89 (-12.57, -1.20)  PFHpS  T1: reference  T3: -4.48 (-9.88, 0.93)  PFNA  T1: reference  T2: 4.45 (-1.30, 10.21)  PFDA  T1: reference  T2: 4.59 (-0.93, 10.11)  (ii) Girls  PFOS  T1: reference  T2: -3.73 (-6.59, -0.87)  PFHxS  T1: reference  T3: -2.22 (-0.37, 3.93)  PFNA  T1: reference  T3: -5.06 (-10.61, 0.48)  PFDA  T1: reference  T2: -3.60 (-9.03, 1.83)  PFHpS  T1: reference  T2: -4.92 (-11.68, 1.85) | Ernst et al. (2019) |
| United Kingdom (1991-1992) | 448 girls (218 cases with menarche < 11.5 years of age vs. 230 control) | Nested Case-control study (ALSPAC) | Maternal (gestational week 16) | Serum | PFOA: 3.7 (2.8-4.8)  PFOS: 19.8 (15.1-24.9)  PFNA: 0.6 (0.5-0.8)  PFHxS: 1.6 (1.2-2.2)  Et-PFOSA-AcOH: 0.6 (0.4-0.9)  Me-PFOSA-AcOH: 0.4 (0.3-0.8) | Earlier age of menarche (< 11.5 years) by questionnaire at 13 years of age | No significant associations were found as single chemical model.  Multi-chemical model  Et-PFOSA-AcOH  OR 1.05 (1.01, 1.10) | Marks KJ et al. (2021) |
| Sex hormone levels | | | | | | | | |
| United Kingdom (1991-1992) | 72 girls | Cohort  (ALSPAC) | Maternal (gestational week 16) | Serum | PFOA: 3.6 (2.7-5.7)  PFOS: 19.2 (15.1-25.0)  PFHxS: 1.6 (1.2-2.1)  PFNA: 0.5 (0.4-0.7) | Total T, SHBG  (At 15 years of age) | (i) Total testosterone (β)  PFOA  T1: reference  T3: 0.24 (0.05, 0.43)  PFOS  T1: reference  T3: 0.18 (0.01, 0.35)  PFHxS  T1: reference  T2: 0.18 (0.00, 0.37)  T3: 0.18 (0.00, 0.35) | Maisonet et al. (2015) |
| United States (2011-2012) | 303 (158 boys, 145 girls) | Cross-sectional (NHANES) | Childhood (12-20 years) | Serum | PFOA (boys/girls): 1.85 (1.46-2.45) / 1.53 (1.15-2.15)  PFOS (boys/girls): 4.60 (3.12-6.88) / 3.76 (2.33-5.57)  PFHxS (boys/girls): 1.28 (0.77-6.88) / 0.83 (0.56-1.74)  PFNA (boys/girls): 0.78 (0.56- 1.19) / 0.73 (0.52-1.10) | T | No significant associations | Lewis et al. (2015) |
| Taiwan (2006-2008) | 95 (30 boys, 65 girls) | Cross-sectional | Childhood (12-17 years) | Serum | PFOA: GM 3.03 (2.93)  PFOS: GM 7.12 (1.95)  PFNA: GM 0.98 (3.53)  PFUnDA: GM 6.42 (3.06) | Total T, free T, E2, SHBG, FSH, LH | (I) Boys  (i) FSH (mean, SE)  PFOS  Q1: 1.50 (0.33)  Q2: 1.56 (0.22) Q3: 1.26 (0.28) Q4: 0.76 (0.29)  (II) Girls  (i) SHBG (mean, SE)  PFOA  Q1: 3.50 (0.24) Q2: 3.50 (0.25) Q3: 3.45 (0.29) Q4: 2.96 (0.34)  (ii) T (mean, SE)  PFOS  Q1: 3.97 (0.23)  Q2: 4.00 (0.23) Q3: 3.87 (0.19) Q4: 3.61 (0.36)  (iii) FSH (mean, SE)  PFUnDA  Q1: 1.56 (0.26)  Q2: 1.59 (0.23)  Q3: 1.37 (0.20) Q4: 1.24 (0.24) | Tsai et al. (2015) |
| Taiwan (2009-2010) | 226 (102 boys, 124 girls) | Cross-sectional | Childhood (13-15 years) | Serum | PFOA: 0.5 (0.4-1.3)  PFOS: 28.9 (14.1-43.0)  PFHxS: 1.3 (0.6-2.8)  PFNA: 0.8 (0.6-1.1)  PFDA: 0.9 (0.8-1.2) PFDoDA: 2.7 (0.8-6.0)  PFTeDA: 5.0 (0.3-23.3)  PFHxA: 0.2 (0.1-0.3)  PFBS: 0.5 (0.4-0.5) | T, E2 | (I) Boys  (i) T (β)  PFOS: -0.0029 (-0.0055, -0.0003)  PFNA: -0.4233 (-0.6998, -0.1467)  PFDA: -0.2565 (-0.4135, -0.9994)  PFHxA: -0.3095 (-0.5942, -0.0248)  (ii) E2 (β)  PFOA: 0.0921 (0.0186, 0.1656)  PFHxS: 0.0462 (0.0020, 0.0905)  (II) Girls  (i) T (β)  PFDA: -0.0119 (-0.0227, -0.0010) | Zhou et al. (2016) |
| United States (2005-2006) | 2292 (1169 boys, 1123 girls) | Cross-sectional (C8 Health Project) | Childhood (6-9 years) | Serum | PFOA: (boys/girls) 34.8 (15.3-82.2) / 30.1 (13.5-74.0)  PFOS: (boys/girls) 22.4 (16.5-32.0) / 20.9 (15.3-29.4)  PFHxS: (boys/girls) 8.1 (4.2-17.1) / 7.0 (3.8-13.8)  PFNA: (boys/girls) 1.7 (1.3-2.3) / 1.7 (1.3-2.4) | Total T, free T, E2 | (I) Boys  (i) T (β)  PFOA: -4.9 (-8.7,-0.8)  PFOS: -5.8 (-9.4, -2.0)  (ii) E2 (β)  PFOS: -4.0 (-7.7, -0.1)  (II) Girls  (i) T (β)  PFOS: -6.6 (-10.1, -2.8) | Lopez-Espinosa et al. (2016) |
| Italy (2017-2018) | 100 boys | Cross-sectional | Adolescents (18-24 years) | Serum | PFOA (exposed/controls) 7.4 (4.7-14.9) / 4.7 (3.5-6.6)  PFOS (exposed/controls): 1.1 (0.8-1.3) / 0.8 (0.4-1.3) | T, FSH, LH | (i) T (Spearman’s correlation coefficient)  PFOA: 0.305  (ii) LH (Spearman’s correlation coefficient)  PFOA: 0224 | Di Nisio et al. (2018) |

IQR, interquartile range; SD, standard deviation; OR, odds ratio; ALSPAC, Avon Longitudinal Study of Parents and Children; PFOA, perfluorooctanoic acid; PFOS, perfluorooctane sulfonic acid; PFHxS, perfluorohexane sulfonic acid; PFNA, perfluorononanoic acid; Et-PFOSA-AcOH, N-ethyl-perfluorooctane sulfonamidoacetic acid; Me-PFOSA-AcOH, N-methyl-perfluorooctane sulfonamidoacetic acid; T, testosterone; E2, estradiol; Q1, 1st quartile; Q2, 2nd quartile; Q3, 3rd quartile; Q4, 4th quartile; T1, 1st tertile; T2, 2nd tertile; T3, third tertile; PFDA, perfluorodecanoic acid; PFHpS, perfluoroheptanane sulfonic acid; SHBG, sex hormone-binding globulin; NHANES, National Health and Nutrition Examination Survey; PFUnDA, perfluoroundecanoic acid; GM, geometric mean; FSH, follicle stimulating hormone; LH, luteinizing hormone; SE, standard error; PFDoDA, perfluorododecanoic acid; PFTeDA, perfluorotetradecanoic acid; PFHxA, perfluorohexanoic acid; PFBS, perfluorobutane sulfonic acid

^*^ Statistically significant findings are described.

Supplementary Table 6. Risk of bias assessment for studies assessing associations between prenatal PFAS exposure and fetal growth

| Study | Risk of bias domain | | | | | | | Overall risk of bias |
| --- | --- | --- | --- | --- | --- | --- | --- | --- |
|  | Confounding | Selection | Classification of exposure | Departure | Missing data | Measurement | Reporting bias |  |
| Apelberg AJ et al. (2007) | Moderate | Low | Low | Low | Low | Low | Low | Moderate |
| Fei C et al. (2007) | Moderate | Low | Low | Low | Low | Low | Low | Moderate |
| Monroy R et al. (2008) | Moderate | Low | Low | Low | Low | Low | Low | Moderate |
| Fei C et al. (2008) | Moderate | Low | Low | Low | Low | Low | Low | Moderate |
| Stein CR et al. (2009) | Serious | Low | Low | Low | Moderate | Serious | Low | Serious |
| Washino N et al. (2009) | Moderate | Low | Low | Low | Low | Low | Low | Moderate |
| Hamim MP et al. (2010) | Serious | Low | Low | Low | Low | Low | Low | Serious |
| Whitworth KW et al. (2011) | Moderate | Low | Low | Low | Low | Low | Low | Moderate |
| Wu K et al. (2012) | Serious | Low | Low | Low | Moderate | Moderate | Low | Serious |
| Chen M-H et al. (2012) | Moderate | Low | Low | Low | Low | Low | Low | Moderate |
| Maisonet M et al. (2012) | Moderate | Low | Low | Low | Low | Low | Low | Moderate |
| Darrow LA et al. (2013) | Moderate | Low | Low | Low | Low | Moderate | Low | Moderate |
| Kishi R et al. (2015) | Serious | Low | Low | Low | Moderate | Low | Low | Serious |
| Bach CC et al. (2016) | Moderate | Low | Low | Low | Low | Low | Low | Moderate |
| Lee E-S et al. (2016) | Serious | Low | Low | Low | Moderate | Low | Low | Serious |
| Alkhalawi E et al. (2016) | Moderate | Low | Low | Low | Low | Low | Low | Moderate |
| Wang Y et al. (2016) | Moderate | Low | Low | Low | Low | Low | Low | Moderate |
| Lenters V et al. (2016) | Moderate | Low | Low | Low | Low | Low | Low | Moderate |
| Sagiv SK et al. (2017) | Moderate | Low | Low | Low | Low | Low | Low | Moderate |
| Manzano-Salgado CB et al. (2017) | Moderate | Low | Low | Low | Low | Low | Low | Moderate |
| Li M et al. (2017) | Serious | Low | Low | Low | Low | Low | Low | Serious |
| Minatoya M et al. (2017) | Moderate | Low | Low | Low | Low | Low | Low | Moderate |
| Shi Y et al. (2017) | Moderate | Low | Low | Low | Low | Low | Low | Moderate |
| Chen M-H et al. (2017) | Moderate | Low | Low | Low | Low | Low | Low | Moderate |
| Woods MM et al. (2017) | Moderate | Low | Low | Low | Low | Low | Low | Moderate |
| Ashley-Martin et al. (2017) | Moderate | Low | Low | Low | Moderate | Low | Low | Moderate |
| Lauritzen et al. (2017) | Moderate | Low | Low | Low | Low | Low | Low | Moderate |
| Meng Q et al. (2018) | Moderate | Low | Low | Low | Low | Low | Low | Moderate |
| Marks KJ et al. (2019) | Moderate | Low | Low | Low | Low | Low | Low | Moderate |
| Eick et al. (2020) | Moderate | Low | Low | Low | Low | Low | Low | Moderate |
| Hjermitslev et al. (2020) | Moderate | Low | Low | Low | Moderate | Low | Low | Moderate |
| Kashino et al. (2020) | Moderate | Low | Low | Low | Moderate | Low | Low | Moderate |
| Souza et al. (2020) | Moderate | Low | Low | Low | Low | Low | Moderate | Moderate |
| Wikström S et al. (2020) | Moderate | Low | Low | Low | Low | Low | Low | Moderate |
| Gardener et al. (2021) | Moderate | Low | Low | Low | Moderate | Low | Low | Moderate |
| Chen et al. (2021) | Moderate | Low | Low | Low | Low | Low | Low | Moderate |
| Lee YJ et al (2013) | Severe | Low | Low | Low | Moderate | Low | Low | Severe |
| Callan AC et al (2016) | Low | Low | Low | Low | Moderate | Severe | Low | Severe |
| de Cock M et al (2016) | Low | Low | Low | Low | Moderate | Low | Low | Moderate |
| Xu C et al (2019) | Moderate | Low | Low | Low | Moderate | Low | Low | Moderate |
| Marks KJ et al (2021) | Low | Low | Low | Low | Moderate | Low | Low | Moderate |
| Hu JMY et al (2021) | Low | Low | Low | Low | Moderate | Low | Low | Moderate |
| Govarts E et al (2016) | Moderate | Low | Low | Low | Moderate | Low | Low | Moderate |
| Rokoff LB et al (2018) | Low | Low | Low | Low | Low | Low | Low | Low |
| Louis et al. (2018) | moderate | Low | Low | Low | Low | Low | Low | Moderate |
| Kalloo et al (2020) | Low | Low | Low | Low | Low | Low | Low | Low |
| Kwon EJ et al (2016) | Moderate | Low | Low | Low | Low | Low | Low | Moderate |
| Kobayashi S et al (2016) | Low | Low | Low | Low | Low | Low | Low | Low |
| Valvi D et al (2017) | Low | Low | Low | Low | Moderate | Low | Low | Moderate |
| Starling AP et al. (2017) | Moderate | Low | Low | Low | Low | Low | Low | Moderate |
| Wang H et al (2019) | Low | Low | Low | Low | Low | Low | Low | Low |
| Xiado C et al. (2020) | Moderate | Low | Low | Low | Low | Low | Low | Moderate |

Supplementary Table 7. Risk of bias assessment for studies assessing associations between PFAS exposure and postnatal growth

| Study | Risk of bias domain | | | | | | | Overall risk of bias |
| --- | --- | --- | --- | --- | --- | --- | --- | --- |
|  | Confounding | Selection | Classification of exposure | Departure | Missing data | Measurement | Reporting bias |  |
| Andersen et al. (2010) | Moderate | Low | Low | Low | Moderate | Low | Low | Moderate |
| Cock et al. (2014) | Moderate | Low | Low | Low | Moderate | Low | Low | Moderate |
| Gyllenhammer et al. (2018) | Low | Low | Low | Low | Low | Low | Low | Low |
| Shoaff et al. (2018) | Low | Low | Low | Low | Low | Low | Low | Low |
| Cao et al. 2018 | Moderate | Low | Low | Low | Low | Low | Low | Moderate |
| Lee et al. (2018) | Low | Low | Low | Low | Low | Low | Low | Low |
| Starling et al. (2019) | Low | Low | Low | Low | Low | Low | Low | Low |
| Tanner et al. (2020) | Moderate | Low | Low | Low | Low | Low | Low | Moderate |
| Scinicariello et al. (2020) | Moderate | Low | Low | Low | Moderate | Low | Low | Moderate |

Supplementary Table 8. Risk of bias assessment for studies assessing associations between PFAS exposure and adiposity

| Study | Risk of bias domain | | | | | | | Overall risk of bias |
| --- | --- | --- | --- | --- | --- | --- | --- | --- |
|  | Confounding | Selection | Classification of exposure | Departure | Missing data | Measurement | Reporting bias |  |
| Halldorsson et al. (2012) | Low | Low | Low | Low | Low | Moderate | Low | Moderate |
| Andersen et al. (2013) | Low | Low | Low | Low | Low | Moderate | Low | Moderate |
| Amalie G et al. (2014) | Moderate | Low | Low | Low | Low | Low | Low | Moderate |
| Høyer et al. (2015) | Low | Low | Low | Low | Low | Moderate | Low | Moderate |
| Braun et al. (2016) | Low | Low | Low | Low | Low | Low | Low | Low |
| Domazet et al. (2016) | Low | Low | Low | Low | Low | Low | Low | Low |
| Mora et al. (2016) | Low | Low | Low | Low | Low | Low | Low | Low |
| Karlsen et al. (2017) | Moderate | Low | Low | Low | Low | Low | Low | Moderate |
| Manzano-Salgado et al. (2017) | Low | Low | Low | Low | Low | Low | Low | Low |
| Hartman et al. (2017) | Moderate | Low | Low | Low | Moderate | Low | Low | Moderate |
| Lauritzen et al. (2018) | Moderate | Low | Low | Low | Low | Low | Low | Moderate |
| Chen et al. (2019) | Moderate | Low | Low | Low | Moderate | Low | Low | Moderate |
| Fassler et al. (2019) | Moderate | Low | Low | Low | Low | Low | Low | Moderate |
| Martinsson et al (2020) | Moderate | Low | Low | Low | Low | Low | Low | Moderate |
| Jensen et al (2020) | Low | Low | Low | Low | Low | Low | Low | Low |
| Liu et al. (2020) | Moderate | Low | Low | Low | Moderate | Low | Low | Moderate |
| Braun et al (2021) | Low | Low | Low | Low | Low | Low | Low | Low |
| Averina et al (2021) | Moderate | Low | Low | Low | Low | Low | Low | Moderate |
| Geiger et al. (2021) | Moderate | Low | Low | Low | Moderate | Low | Low | Moderate |

Supplementary Table 9. Risk of bias assessment for studies assessing associations between PFAS exposure and puberty, menarche, and sex hormone levels

| Study | Risk of bias domain | | | | | | | Overall risk of bias |
| --- | --- | --- | --- | --- | --- | --- | --- | --- |
|  | Confounding | Selection | Classification of exposure | Departure | Missing data | Measurement | Reporting bias |  |
| Puberty, menarche | | | | | | | | |
| Christensen et al. (2011) | Moderate | Low | Low | Low | Low | Low | low | Moderate |
| Lopez-Espinosa et al. (2011) | Serious | Low | Moderate | Low | Moderate | Low | Moderate | Serious |
| Kristensen et al. (2013) | Serious | Low | Low | Low | Moderate | Low | Low | Serious |
| Ernst et al. (2019) | Moderate | Low | Low | Low | Moderate | Low | Low | Moderate |
| Marks KJ et al. (2021) | Moderate | Low | Low | Low | Low | Low | Low | Moderate |
| Sex hormone levels | | | | | | | | |
| Maisonet et al. (2015) | Moderate | Low | Low | Low | Moderate | Low | Low | Moderate |
| Lewis et al. (2015) | Moderate | Low | Low | Low | Moderate | Low | Low | Moderate |
| Tsai et al. (2015) | Moderate | Low | Low | Low | Moderate | Low | Moderate | Moderate |
| Zhou et al. (2016) | Moderate | Low | Low | Low | Low | Low | Low | Moderate |
| Lopez-Espinosa et al. (2016) | Moderate | Low | Low | Low | Moderate | Low | Low | Moderate |
| Di Nisio et al. (2018) | Serious | Low | Low | Low | Moderate | Low | Low | Serious |

**Supplementary Figure legends**

Supplementary Figure 1. Flow chart of the study selection process
