# Supplementary figures and images for: Early-Life Exposure to Per- and Poly-Fluorinated Alkyl Substances and Growth, Adiposity, and Puberty in Children: A Systematic Review
Source: Front Endocrinol (Lausanne). 2021 Sep 9;12:683297. doi: 10.3389/fendo.2021.683297 (PMC8458955; doi:10.3389/fendo.2021.683297)

Supplementary Figure 1

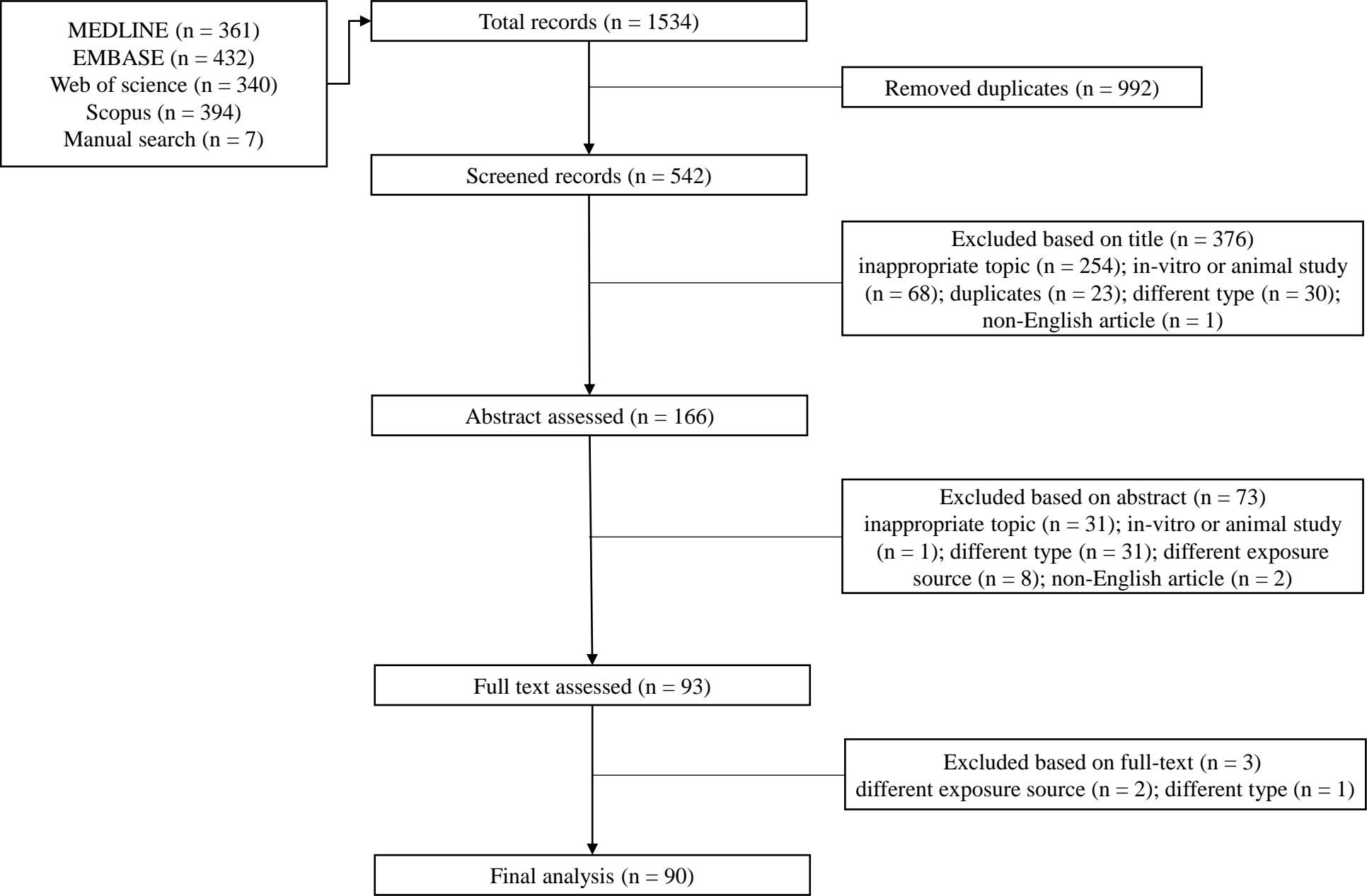

Supplement: Supplementary file 1 [file DataSheet_1.zip › Supplementary Figure 1.PDF]
